# Insight into PreImplantation Factor (PIF*) Mechanism for Embryo Protection and Development: Target Oxidative Stress and Protein Misfolding (PDI and HSP) through Essential RIPK Binding Site

**DOI:** 10.1371/journal.pone.0100263

**Published:** 2014-07-01

**Authors:** Eytan R. Barnea, David M. Lubman, Yan-Hui Liu, Victor Absalon-Medina, Soren Hayrabedyan, Krassimira Todorova, Robert O. Gilbert, Joy Guingab, Timothy J. Barder

**Affiliations:** 1 Research & Development, SIEP The Society for the Investigation of Early Pregnancy, Cherry Hill, New Jersey, United States of America; 2 Research & Development, BioIncept, LLC, Cherry Hill, New Jersey, United States of America; 3 Department of Obstetrics, Gynecology and Reproduction, UMDNJ-Robert Wood Johnson Medical School, Camden, New Jersey, United States of America; 4 Department Surgery, University of Michigan Medical Center, Ann Arbor, Michigan, United States of America; 5 Reproductive Medicine, Cornell University, College of Veterinary Medicine, Ithaca, New York, United States of America; 6 Institute of Biology and Immunology of Reproduction, Bulgarian Academy of Sciences, Sofia, Bulgaria; 7 Chemical Biology and Proteomics, Banyan Biomarkers, Alachua, Florida, United States of America; 8 Research & Development, Eprogen, Downers Grove, Illinois, United States of America; Institute for Virus Research, Laboratory of Infection and Prevention, Japan

## Abstract

**Background:**

Endogenous PIF, upon which embryo development is dependent, is secreted only by viable mammalian embryos, and absent in non-viable ones. Synthetic PIF (sPIF) administration promotes singly cultured embryos development and protects against their demise caused by embryo-toxic serum. To identify and characterize critical sPIF-embryo protein interactions novel biochemical and bio-analytical methods were specifically devised.

**Methods:**

FITC-PIF uptake/binding by cultured murine and equine embryos was examined and compared with scrambled FITC-PIF (control). Murine embryo (d10) lysates were fractionated by reversed-phase HPLC, fractions printed onto microarray slides and probed with Biotin-PIF, IDE and Kv1.3 antibodies, using fluorescence detection. sPIF-based affinity column was developed to extract and identify PIF-protein interactions from lysates using peptide mass spectrometry (LC/MS/MS). *In silico* evaluation examined binding of PIF to critical targets, using mutation analysis.

**Results:**

PIF directly targets viable cultured embryos as compared with control peptide, which failed to bind. Multistep Biotin-PIF targets were confirmed by single-step PIF-affinity column based isolation. PIF binds protein disulfide isomerases a prolyl-4-hydroxylase β-subunit, (PDI, PDIA4, PDIA6-like) containing the antioxidant thioredoxin domain. PIF also binds protective heat shock proteins (70&90), co-chaperone, BAG-3. Remarkably, PIF targets a common RIPK site in PDI and HSP proteins. Further, single PIF amino acid mutation significantly reduced peptide-protein target bonding. PIF binds promiscuous tubulins, neuron backbones and ACTA-1,2 visceral proteins. Significant anti-IDE, while limited anti-Kv1.3b antibody-binding to Biotin-PIF positive lysates HPLC fractions were documented.

**Conclusion:**

Collectively, data identifies PIF shared targets on PDI and HSP in the embryo. Such are known to play a critical role in protecting against oxidative stress and protein misfolding. PIF-affinity-column is a novel utilitarian method for small molecule targets direct identification. Data reveals and completes the understanding of mechanisms involved in PIF-induced autotrophic and protective effects on the embryo.

## Introduction

Shortly after fertilization the embryo/allograft is surrounded by the zona-pellucida which physically separates the embryo from the maternal environment. While maternal-derived compounds still can reach the embryo, access through the embryo cell membrane is more limited. It favors transfer of lipophilic, cationic and large conjugated system compounds [1]. Maternally-derived or embryo-secreted trophic compounds were previously reported. They include transforming growth factor-α, GNRH I analog, insulin growth factors, acrogranin, epidermal growth factor, embryotrophic factor 3 and GMCSF among others [2]–[10].

The Barnea research group focuses on those compounds involved in the intimate and essential embryo-maternal cross-talk that initiates shortly post-fertilization and continues throughout viable pregnancy. Specifically, PreImplantation Factor (PIF) is an embryo-specific fifteen amino acid linear peptide, secreted only by viable embryos and absent in non-viable ones [11]–[19]. In culture, PIF is secreted already by the two-cell stage mouse embryo, in four-cell stage human embryos, and in bovine embryos at the six-cell stage [18], [19]. Increased PIF levels in culture correlate with embryo progress to the blastocyst stage [19]. PIF is expressed by the placenta and hemopoietic fetal tissues and is detected in maternal circulation throughout gestation [20], [21]. Detection of PIF already at day 20 post- artificial insemination correlates highly with favorable pregnancy outcome. Exogenous sPIF (synthetic identical structure) administered to singly cultured bovine embryos, enhances their development to the blastocyst stage [22]. The inhibitory effect of anti-PIF monoclonal antibody added in murine culture documents the critical autotrophic role that PIF exerts on embryo survival [19]. PIF acts as a rescue factor, protecting cultured murine embryos against toxicity of serum (i.e. containing oxygen radicals, antibodies) derived from patients with a history of recurrent pregnancy loss (RPL) [22]. Beyond supporting embryo development, PIF also regulates the maternal environment by promoting embryo receptivity acting on endometrium, implantation phase and first trimester decidua [23], [24]. PIF also promotes trophoblast invasion, and orchestrates maternal systemic immune response [18], [25], [26]. The embryo may be protected by reducing systemic NK cells' toxicity of patients with a history of RPL [26]. Pathway analysis in autoimmunity and transplantation models demonstrate that sPIF, administered as a single agent to non-pregnant mice, acts by reducing oxidative stress and protein misfolding [27]–[31]. ProtoArray data reveals that PIF primarily targets insulin degrading enzyme (IDE) and Kv1.3b potassium channel proteins [23]. While detailed studies in the decidua and on systemic immunity have already identified mechanisms involved in PIF's targeted action, no such information is available on the identity of the specific binding sites of PIF in the embryo.

Such data would help to identify those sites that are involved in embryo protection and development. The objective of this study is to determine PIF specific targets within the embryo. This was carried out by using both cultured murine and equine embryos and biochemical methods. Complementary isolation methods in murine embryo extracts were followed using liquid chromatography tandem mass spectrometry (LC/MS/MS) and immunohistochemistry. In silico design followed by mutation analysis confirmed binding site in protein targets. We report that the specific PIF targets identified PDI/HSP could have important supportive and protective roles in embryo development regulating oxidative stress and protein misfolding.

## Materials and Methods

### PIF Peptide Synthesis and Labeling

Synthetic PIF (MVRIKPGSANKPSDD) and scrambled PIF (GRVDPSNKSMPKDIA), used as control, were obtained by solid-phase peptide synthesis (Peptide Synthesizer, Applied Biosystems, Foster City, CA) using Fmoc (9-fluorenylmethoxycarbonyl) chemistry. The same peptides were also labeled with FITC on their N-termini in the solid phase after the addition of l-alanine as a spacer group. Final purification was conducted by reversed-phase HPLC and their identity was verified by MALDI-TOF mass spectrometry and amino acid analysis. Biotin conjugated PIF was also generated at Biosynthesis, Inc. (Lewisville, TX).

### FITC-PIF Binding to Embryos (Murine)

The study has been approved by Cari Research Institute, Chicago, IL. A routinely used mouse embryo culture procedure recently published was performed in this study [19]. Two-cell murine embryos were cultured up to blastocyst stage and subsequently exposed to 5–25 µg/ml of FITC-PIF or FITC-scrambled PIF for 30 min in a dark chamber. Embryos were subsequently washed in PBS to remove non-specific binding and then fixed with 1.5% formaldehyde in PBS. The prepared slides were examined under a fluorescent microscope fitted with a digital camera, 40× magnification located at the Northwestern University core facility, (Chicago, IL).

### FITC-PIF Up-take by Blastocysts (Equine)

The equine blastocyst retrieved from pregnant mares is sufficiently large to enable examination of an advanced embryo where fertilization has occurred in vivo. The study has been approved by Cornell University, College of Veterinary Medicine. Seven days after ovulation, 2 embryos were flushed from 2 different mares and cultured for 30 min in synthetic oviductal media droplets containing 5 µg/ml FITC-PIF or FITC-PIF scrambled in a humidified atmosphere, 5% CO_2_, 7% O_2_ and balanced N_2_ with a temperature 38.5°C. Subsequently, blastocysts were washed in 1× PBS with 0.1% PVP to remove non-specific binding and fixed with 4% paraformaldehyde containing 0.1% triton x-100. All washings and fixation steps were carried out in 96-well dishes. After 3 more washes to remove fixative solution, embryos were mounted on slides with two etched 10 mm diameter circles surrounded by white ceramic ink. Finally, embryos were covered with ProLong Gold Antifade Reagent with DAPI (Life Technologies, Eugene, Oregon, USA).

### Image Acquisition

All slides were visualized using a microscope (Imager Z1; Carl Zeiss, Inc.) under a 20×0.5 NA ECPlan Neofluar air immersion (Carl Zeiss, Inc.). The fluorochromes used FITC and nuclei were observed using DAPI filter. Slides were excited at 340 nm and 488 nm to visualize DAPI nuclear stain and FITC, respectively. Images were captured with a cooled charged-coupled device camera (AxioCam MRm; Carl Zeiss, Inc.) and processed using AxioVision software (version 4.7.2; Carl Zeiss, Inc.). Images were taken as superimposed (FITC/DAPI), FITC and DAPI, alone. Seven days after ovulation, equine embryos were too large to fit a 20× magnification. Therefore, pictures were taken in sections and subsequently joined.

### Embryo Lysate Fractionations (10 Day Old Murine)

The 10-day old mouse embryo transitions from embryonic to the fetal stage, at the time when neural tube fuses, cardiovascular and cervical inter-somitic vessels form and connect, as well as forelimb buds start to develop [32]. Therefore, this model provides a means to elucidate PIF-embryo direct interaction. In addition, 10-day old mouse embryo extracts provide useful sources of potential PIF targets that can be easily examined using modern proteomics tools such as protein microarrays and PIF-based affinity chromatography and liquid chromatography tandem mass spectrometry (LC/MS/MS).

2.5 mg of 10 day old mouse embryos lysates (5 vials) (Zyagen) were diluted to 2.5 ml with ProteoSep Start Buffer (SB) pH 8.6 (Eprogen) and buffer exchanged with 3.5 ml of SB using a PD-10 column (GE Healthcare) according to manufacturer's instructions. The PD-10 eluent was diluted to 5.0 ml with SB and 1.0 ml aliquots were placed into 5 separate wells of a 1.8 ml/well 96-well plate. Using a ProteoSep HPRP HPLC column (Eprogen), 500 µl of the PD-10 exchanged lysates were separated and fractions collected using a ProteomeLab PF2D HPLC instrument under the following conditions:

Mobile Phase A: H_2_O/0.1% TFA; Mobile Phase B: Acetonitrile/0.08% TFA

Gradient: 0–5% B in 2 min, 5–15% B in 1 min, 15–25% B in 2 min, 25–30% B in 3 min, 30–41%B in 15 min, 41–47% B in 4 min, 47–67% B in 5 min, 67–100% B in 3 min. Injection volume: 250 µl, Temperature: 50°C, Flow rate: 750 µl/min, Detection: UV 214 nm. Fractions from the HPRP analysis were collected into 0.65 ml/well 96 well plates (Orochem) using a Gilson FC 204 fraction collector from 5–40 min @ 0.37 min/well. Each well contained 276 µl with 96 wells collected per fractionation. The plates were covered with foil sealing tape (3M) and stored at −80°C until further use.

### Microarray Preparation

50 µl of a 40% glycerol (Sigma ultra-pure) water solution was added to each well of 96-well plates and then evaporated down to a final volume of 20 µl using a SpeedVac (ThermoFisher). 30 µl of PBS was then added to each well to make a 40% glycerol/PBS print buffer and the contents of each well transferred into 2 sets of 384 well plates for microarray printing. A solid (150 um) pin QArray2 (Genetix, Ltd.) was used to spot the arrays on thin layer nitrocellulose PATH slides (Gentel Biosciences, Inc.). Each well was double spotted and 5 sets of arrays of the 96 wells fractions collected were printed on PATH slides with 2 printings on each slide. Positive and negative controls were printed along with the fractions using. Slides were stored in a sealed slide box until used.

### Microarray Assay

The printed slides were blocked in 1% BSA in PBS+0.5% Tween 20 for 1 hr and then rinsed with PBS+0.5% Tween 20. Goat Anti-Human-IDE or Anti-Kv1.3b channel antibody (R&D Systems) was incubated at 1, 5 and 10 µg/ml in 1% BSA in PBS+0.5% Tween 20 for 2 hrs. The slides were washed 3 times in PBS+0.5% Tween 20, incubated with Chicken Anti-Goat IgG Alexa 647 for the Anti-IDE and anti-Kv1.3b for 1 hr, washed 3 times in PBS+0.5% Tween 20 and then rinse briefly in ddH2O. Biotin labeled PIF binding was determined by using streptavidin labeled Alexafluor-647 fluorescence dye using the average of the intensities of the spots and scanned using a Genepix scanner.

### Protein Identification by LC/MS/MS

The selected protein fractions were digested with trypsin at 37°C overnight. The resulting peptides were analyzed by LC/MS/MS using an LTQ mass spectrometer (Thermo Finnigan, San Jose, CA). Chromatographic separation of peptides was performed on a Paradigm MG4 micropump system (Michrom Biosciences Inc., Auburn, CA) equipped with a C18 separation column (0.1 mm×150 mm, C18 AQ particles, 5 µm, 120 Å, Michrom Biosciences Inc., Auburn, CA). Peptides were separated with a linear gradient of acetonitrile/water containing 0.1% formic acid at a flow rate of 300 nl/min. A 120 minute linear gradient separation was used. The MS instrument was operated in positive ion mode. The ESI spray voltage was set at 2.5 kV and the capillary voltage at 30 V. The ion activation was achieved by utilizing helium at a normalized collision energy of 35%. The data were acquired in data-dependent mode using the Xcaliber software. For each cycle of one full mass scan (range of m/z 400–2000), the three most intense ions in the spectrum were selected for tandem MS analysis, unless they appeared in the dynamic or mass exclusion lists.

### Data Analysis

All MS/MS spectra were searched against the IPI database (IPI.mouse.v3.79). The search was performed using SEQUEST algorithm version 27 incorporated in Bioworks software version 3.1 SR1 (Thermo Finnigan). The search parameters were as follows: (1) Fixed modification, Carbamidomethyl of C; (2) variable modification, oxidation of M; (3) allowing two missed cleavages; (4) peptide ion mass tolerance 1.50 Da; (5) fragment ion mass tolerance 0.0 Da; (6) peptide charges +1, +2, and +3. The identified peptides were processed by the Trans-Proteomic Pipeline (TPP) [33]. This software includes both the PeptideProphet and ProteinProphet programs. The database search results were first confirmed using the PeptideProphet software, and then the peptides were assigned for protein identification using the ProteinProphet software. In this study, both the PeptideProphet probability score and the ProteinProphet probability score were set to be higher than 0.9. This resulted in an overall false positive rate below 1% [34].

### PIF-resin Affinity Chromatography (Specially Designed)

A PIF-resin affinity column was specifically designed for this study, to replace the commonly used multistep method. Briefly, to PIF 15AA) a carbon spacer (C6) at N-terminus following with a Cysteine at the end and then the thiol group of the cysteine was conjugated to agarose resin (Biosynthesis, TX). The protocol for extractions of Zyagen 10 day old embryos was as follows: 25 µl of Resin centrifuged for 1 min (6,000× g) and washed 2× with 100 µl nondetergent lysing buffer (NDLB) (Eprogen) in a compact reaction tube (Becton-Dickenson). A vial of Zyagen Lysate (∼100 µl, 0.1 mg total protein) was added to the washed resin and incubated 1 hr at RT. The tubes were centrifuged for 1 min (6000× g) and washed 2× with 100 µl NDLB. Filtrates were combined and diluted to 400 µl total volume for HPLC runs.

To the treated resin, 200 µl of 0.1M Glycine-HCl solution added and agitated for 10 min and then centrifuged for 1 min. The resin was then washed with 200 µl of the 0.1M Glycine-HCl solution and centrifuged for 1 min at RT. The 4 solutions were placed into a deep well 96 well plate and fractionated run using the ProteoSep HPRP column on the 2D HPLC instrument. 250 µl Injections, UV214 nm, 750 µl/min, T = 50°C. ProteoVue software (Eprogen) was used to aid in the analysis and visulaization of the chromatograms.

### LC/MS/MS Analysis

#### Trypsin Digestion

In-solution trypsin digestion of the protein extracts was conducted using the Filter-Assisted Sample Preparation digestion kit (FASP) according to the manufacturer's procedure (Protein Discovery, Expedeon, San Diego, CA). Briefly, 40 µl of protein extract was reduced with 4 µmol DTT at room temperature for 1 hr. The sample was mixed with 200 µl of urea sample solution in the spin filter and centrifuged at 14,000× g for 15 min. Sample flow through was discarded after washing with another 200 µl of urea sample solution. Proteins on the spin filter were alkylated with iodoacetamide in 90 µl urea sample solution for 20 min in the dark. The proteins in the filter were washed twice with 100 µl urea sample solution and centrifuged at 14, 000× g for 10 min. Then, 100 µl of 50 mM ammonium bicarbonate (NH_4_HCO_3_) was added to the spin filter and centrifuged at 14,000× g for 10 min and repeated twice. Trypsin digestion was conducted at 37°C overnight using a trypsin∶protein ratio of 1∶100. After incubation, the spin filter was washed twice with 40 µl of 50 mM NH_4_HCO_3_ and centrifuging at 14,000× g for 10 min, collecting the filtrate into a clean tube. Peptides were extracted by adding 50 µl of 0.5 M sodium chloride solution and centrifuging at 14,000× g for 10 min. The collected filtrate containing the tryptic peptides was acidified with 5 µl formic acid and desalted via C18 solid phase extraction (SPE) (Supelco Discovery SPE, Sigma Aldrich, Saint Louis, MO). The filtrate was dried under vacuum and tryptic peptides were resuspended in 20 µl 0.1% formic acid for subsequent LC/MS/MS analysis.

#### LC/MS/MS analysis

The samples were analyzed by reversed phase nanoflow liquid chromatography and tandem mass spectrometry (LC-MS/MS) using an Easy nLC-II system (Thermo, San Jose, CA) coupled to a Thermo LTQ Velos dual pressure linear ion trap system (Thermo, San Jose, CA). Standard equine cytochrome C digest was injected as a quality control. Two microliters sample was loaded via the autosampler onto a trap column (EASY-Column 2 cm, ID 100 µm, 5 µm, C18-A) then directed to an analytical column (EASY-Column, 10 cm, ID 75 µm, 3 µm, C18-A2) at a flow rate of 250 nl/min. The mobile phase consisted of solvent A (99.9% water with 0.1% formic acid) and solvent B (99.9% acetonitrile with 0.1% formic acid). Separation was achieved using a run time of 100 min. The first linear gradient was from 2% to 40% B over 90 min, the second linear gradient was from 40% to 80% B over 5 min and held for 5 min before returning to the initial mobile phase composition (2% B). Tandem mass spectra (MS/MS) were acquired on the top 10 most abundant ions at a given chromatographic time point by data-dependent scanning.

#### Peptide/Protein Identification

All tandem mass spectra were extracted by Xcalibur version 2.7.0 and analyzed by Sequest (Proteome Discoverer, Thermo, San Jose, CA) and X! Tandem (www.thegpm.org;version 2007.01.01.1). Sequest (v. 1.3.0.339) and X! Tandem were set up to search a trypsin-indexed reversed concatenated IPI mouse protein database (v3.86, 119068 entries) with a fragment ion mass tolerance of 0.8 Da and a parent ion tolerance of 2.0 Da. Carbamidomethylation of cysteine was specified in Sequest and X! Tandem as a fixed modification and oxidation of methionine was as variable modification. Scaffold 3 (ProteomeSoftware, Portland, OR) was used to compile Sequest search results and validate MS/MS based peptide and protein identifications [33]. Peptide identifications were accepted if they could be established at greater than 95.0% probability as specified by the Peptide Prophet algorithm [33]. Protein identifications was accepted if they could be established at greater than 99.9% probability and contained at least 2 identified peptides. Protein probabilities were assigned by the Protein Prophet algorithm [34]. Label-free relative abundance quantitation was done by spectral counting approach.

### Alexa-PIF Interaction with Human Proteome Array

ProtoArray Human Protein Microarrays v5.0 (Invitrogen, Carlsbad, CA) having 9000 possible targets were initially blocked and then exposed to PIF-Alexa 647 (250 nM and 2.5 µM). Unbound probe was washed off and arrays read using a fluorescence microarray scanner. As a negative control, an array was incubated with wash buffer while calmodulin kinase was used as positive control detected by AlexaFluor 647-conjugated anti-v.5 antibody. Three stringent conditions determined AlexaFluor-PIF specificity of binding; signal intensity >200 relative fluorescent units, >6 time higher than the negative control, and Z-factor >5.

### Modeling *in silico* of PIF binding, single amino acid mutagenesis and validation

Using *de novo* peptide structure prediction wild type PIF_1-15_ and several PIF_1-15_ mutant structural models were created by the server PEP-FOLD (http://bioserv.rpbs.univ-paris-diderot.fr/PEP-FOLD/) based on their primary structure (amino-acid sequence) alone [35], [36].

Subsequently the highest ranking PIF binding partners identified in embryo extracts namely PDI and HSP were further analyzed. These proteins were subject to *in silico* assessment of the potential binding of PIF to their protein surfaces. This was assessed in terms of probability, PIF participating residues, targeted protein ligand-receptor surface determining residues using the PepSite 2 server (http://pepsite2.russelllab.org/). We designed an automated Taverna (www.taverna.org.uk) workflow to query PepSite 2 REST service by sending selected targets PDB and PIF_1-10_ for binding score, probability and residue positions of PIF and their binding to corresponding residue positions at the protein targets (**Table S5 in File S1**). Only the first 10 PIF amino acids were used, as it was shown previously that the PIF_1-9_ and the PIF_1-15_ form both share same biological functionality.

High-resolution peptide docking of PIF to its potential targets was done using Rosetta FlexPepDock server (http://flexpepdock.furmanlab.cs.huji.ac.il/). A PDB encoded model of the de novo PIF model was supplied in close proximity and its location was specified by the PepSite 2. For the protein targets PDI and HSP binding PIF structure was predicted *ab initio* using the Pep-Fold server (http://bioserv.rpbs.univ-paris-diderot.fr/PEP-FOLD/). FlexPepDock allows full flexibility to the peptide and side-chain flexibility to the target protein, thus providing accurate refinement of the peptide structure, starting from up to 5.5 RMSD of the native conformation. (RMSD - root-mean-square deviation, is the measure of the average distance between the atoms [usually the backbone atoms] of superimposed proteins) [37].

An analysis of PIF specificity of target binding interface was done using the BeAtMuSiC server (http://babylone.ulb.ac.be/beatmusic/). We used a single aa *in silico* mutagenesis and validation to estimate interface energy change generated by mutated PIF *de novo* models (PepFold). Re-docking by FlexPepDock was compared with the initial PIF-target docking model as a reference. The estimated bbRBMS (Root Mean Square Deviation of peptide backbone heavy atoms only) change between wt PIF and mutated PIF docking models and their Interface energy were compared for the highest Total Rosetta energy per model. This determines the total energy of the PIF-protein complex less the energy of the binding partners when they are separated.

### Statistical Analysis

Chi square was used to analyze the antibody binding data.

## Results

### FITC-PIF Uptake by Cultured Blastocysts (murine)

**Figure 1:**
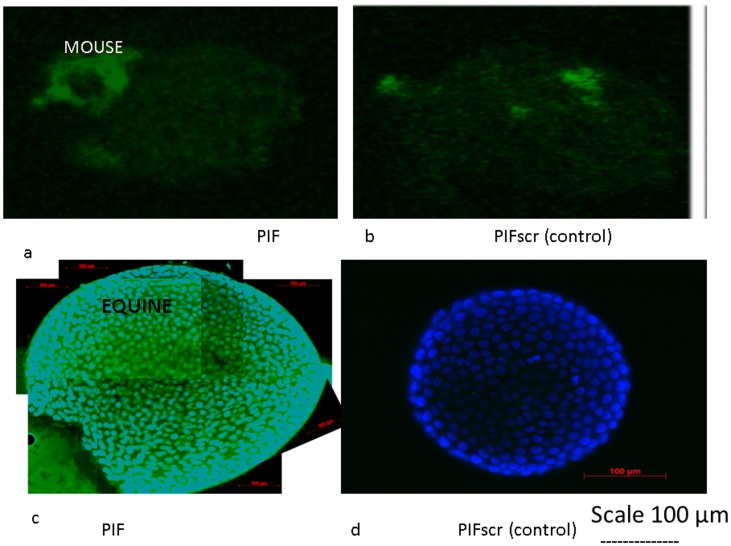
PIF up-take is specific binds only viable mammalian embryos. Mouse embryo blastocysts were cultured in the presence of (**a**) FITC-PIF or (**b**) FITC-PIF scrambled (control). PIF was taken up only by the active peptide - fluorescent microscopy analysis. Equine blastocysts were cultured in the presence of (**c**) FITC-PIF or (**d**) FITC-PIF scrambled (control). Blastocysts were counterstained with DAPI (nuclear stain). Data demonstrated that PIF is only taken up by blastocysts with intact nuclei.

To document endogenous PIF's supporting role, the uptake of FITC-PIF by cultured mouse blastocysts using a fluorescent microscope was examined. FITC-PIF added at 5 µg/ml concentration to the culture medium was taken up and detected within the embryoblast following a short-term culture. (**Fig. 1a**). Observed fluorescence was mostly located at the embryoblast region of the blastocyst. In contrast, the same or even four-fold higher concentration of the scrambled FITC-PIF which was tested in parallel as control did not show any detectable binding to the blastocyst. (**Fig. 1b**).

### FITC-PIF Up-take by Cultured Blastocysts. (equine)

Using mouse blastocysts, we documented that PIF uptake is specific. However, we aimed to document that it is an active uptake which takes place only in viable and not in non-viable embryos. Using the equine model where the size of the blastocysts is suitably large, a better view of labeled PIF could be visualized. In equine blastocysts cultured in the presence of FITC-PIF significant uptake of the peptide was noted (**Fig. 1c**). In contrast, scrambled FITC-PIF (control) failed to bind (**Fig. 1d**). Using DAPI counterstaining enabled us to document that these blastocysts' nuclei were intact in both cultures. This demonstrates that PIF targets the embryo through an active process and not by passive diffusion. Also that only the native and not the scrambled the peptide targets the embryo- indicating binding specificity.

### PIF Targets a Limited Number of Fractions of Embryo Extracts

Having demonstrated that PIF uptake is specific, and the scrambled PIF failed to bind, the aim was to enhance the definition of the specific targets involved. For this end, the 10-day old mouse embryo was used to evaluate PIF targets. To assure purity of the embryonic tissue the possibility of contamination by albumin was addressed. We found that contamination by albumin was minimal, <5% (Western blot, data not shown). Subsequently, embryos were extracted and further separated to reduce complexity of protein mixture present by using reversed phase HPLC based separation. This was followed by printing (spotting) of a small portion of the separated liquid fractions onto nitrocellulose coated microarray slides (**Figure S1 and Figure S2a,b**). The microarrays were then probed with Biotin-PIF, treated with streptavidin Alexafluor 647 fluorescence and analyzed. Biotin-PIF was found to bind in a significant manner to only to a limited number of spots 6 out of 96 (**Figure S3**) indicating that binding of PIF is highly selective in the embryo. (**Figure S4**)

Once a spot was identified as being PIF-positive its binding specificity was validated by using several additional slides (∼5). This methodology of HPLC fractionation to separate complex mixture of proteins followed by microarray analysis enables a simple and reproducible means of isolating specific PIF binding partners. This method permits separation of complex mixtures, enables determination of their respective coverage (estimated concentration) and also establishes a given protein ranking within the extract.

The confirmed limited PIF positive (6/96) fractions were then further analyzed by using LC/MS/MS. **(** **Tables 1** **–** **3** **) and (Table S1–S3 in File S1)** detail the proteins identified by LC/MS/MS in the various PIF-interacting fractions from the microarray analysis. The highest ranking proteins identified are determined according to the number of peptides observed reflecting their relative abundance in the fraction.

**Table 1:** Biotin-PIF binds the G5 fraction in mouse embryo extracts.

Description	Accession	Coverage	# Peptides	# AAs	MW [kDa]	calc. pI	Score
Stress-induced-phosphoprotein 1 STIP1	IPI:IPI00121514.3	21.18	90	543	62.5	6.80	428.34
Peptidyl-prolyl cis-trans isomerase Pin1	IPI:IPI00554989.3	16.77	58	167	18.3	7.90	261.57
Serum albumin	IPI:IPI00131695.3	8.88	46	608	68.6	6.07	229.92
BAG3	IPI:IPI00331334.3	9.01	25	577	61.8	7.44	113.12
40S ribosomal protein S3a	IPI:IPI00331345.5	6.44	13	264	29.9	9.73	86.92
Isoform 2 of Fibulin-2 IFBLN2	IPI:IPI00750260.1	0.77	11	1174	126.4	4.65	50.51
Thioredoxin	IPI:IPI00226993.5	26.67	11	105	11.7	4.92	45.45

**Table 2:** Biotin-PIF binds the H5 fraction in mouse embryo extracts.

Description	Accession	Coverage	# Peptides	# AAs	MW [kDa]	calc. pI	Score
Thioredoxin	IPI:IPI00226993.5	8.57	6	105	11.7	4.92	26.72
zinc finger factor Zbtb45	IPI:IPI00284393.4	2.12	1	520	54.9	7.01	1.79
Isoform 2 of Polycomb protein SCMH1	IPI:IPI00125562.1	1.66	1	664	73.7	9.32	1.77

**Table 3:** Biotin-PIF binds the F6 fraction in mouse embryo extracts.

Description	Accession	Coverage	# Peptides	# AAs	MW [kDa]	calc. pI	Score
Iso 2Tropomyosin a-3	IPI:IPI00230044.5	41.53	44	248	29.0	4.78	238.27
SET translocation	IPI:IPI00755843.1	24.19	21	215	24.9	5.55	139.50
Thioredoxin	IPI:IPI00226993.5	22.86	11	105	11.7	4.92	65.55
Isoform C of Lamin-A/C	IPI:IPI00400300.1	12.20	11	574	65.4	6.80	101.53
DnaJ homolog B11	IPI:IPI00320241.1	7.54	10	358	40.5	6.32	43.94
Nucleolin	IPI:IPI00317794.5	8.77	8	707	76.7	4.75	79.28
Eef1b	IPI:IPI00320208.3	12.44	7	225	24.7	4.69	33.18
Eef1d	IPI:IPI00831299.2	18.60	7	129	14.2	5.87	30.47
Aldoa	IPI:IPI00856415.1	10.69	7	131	14.2	8.15	40.73
Transthyretin	IPI:IPI00127560.1	19.05	6	147	15.8	6.16	38.73
Granzyme G	IPI:IPI00120421.1	5.24	5	248	27.4	9.57	37.01
Zinc finger protein 2	IPI:IPI00222204.5	3.28	5	427	48.6	8.79	135.80
Carbonic anhydrase 1	IPI:IPI00230320.6	7.28	5	261	28.3	6.96	30.46
AHNAK isoform 1	IPI:IPI00553798.2	5.66	5	5656	###	6.30	66.48

### Thioredoxin (TRX) and Related Proteins as Possible PIF Binding Partners: Role in Control of Oxidative Stress

The MS profile of the PIF-bound fractions showed in 2 cases [G5 fraction (**Table 1**) and H5 (**Table 2**)] that the TRX enzyme had the highest coverage. In the F6 fraction (**Table 3**), TRX also had a high coverage and ranked third. These data clearly point to TRX as the most abundant protein within the PIF positive fractions. Moreover, TRX-dependent peroxide reductase (PRDX4) was identified in the G12 fraction (**Table S1 in File S1**) and found to have highest coverage in that fraction. In addition, Pin1 was also observed a peptidyl-prolyl cis-trans isomerase that has anti-apoptotic activity and blocks oxidative stress by reducing nitric oxide formation [38].

### Heat-shock Proteins (HSPs) as Binding Partners for PIF: Role in Regulation of Protein Misfolding

In a related manner, a number of additional proteins beyond TRX were identified as potential targets for PIF. These were observed in the G5 fraction (**Table 1**) and belong to the highly abundant heat-shock protein (HSP) family, well known for their protective properties and involvement in cell repair and signal transduction. Among those identified were STIP1 which mediates association of chaperones HSP70 and HSP90 and BAG3 which inhibits the chaperone activity of HSP70/HSP90 by promoting substrate release.

### Tubulins as PIF Binding Partners: Role in Promotion of Neural Development

The MS analysis of the B9 fraction (**Table S2 in File S1**) identified several neural elements, specifically tubulins, the backbones of neurons. Among those identified were tubulin 2C and b-5 along with four additional tubulins in lower abundance. In the H5 fraction (**Table 2**), two additional proteins involved in neural development were also identified; zinc finger factor (Zbtb45) and SCMH1 which, in 10-day old mouse embryos, is strongly expressed in several parts of the nervous system and visceral organs.

### Actins as PIF Binding Partners: Role in Promotion Vascular and Visceral Development

In the A9 fraction (**Table S3 in File S1**), the highest coverage protein identified was actin (ACTA2), an ATP binding site protein involved in embryo vascularization. In the B9 fraction (**Table S2 in File S1**), Profilin was detected - an actin binder, ADP-ribosylation factor 1 - involved in actin remodeling, as well as four other actins and a light chain myosin. The protein Eef1a1, a promoter of GTP-dependent aminoacyl-tRNA that binds to ribosomes required for effective protein synthesis was identified along with CES2 which detoxifies xenobiotics in the liver. In the G12 fraction (**Table S1 in File S1**) Scx protein was identified which plays an essential role in early embryo mesoderm formation and formation of somite-derived chondrogenic lineages.

### PIF-Affinity Chromatography Extraction Method: Correlation to, and Confirmation of, PIF Binding Partners Identified by Biotin-PIF Positive Factions

**Figure 2:**
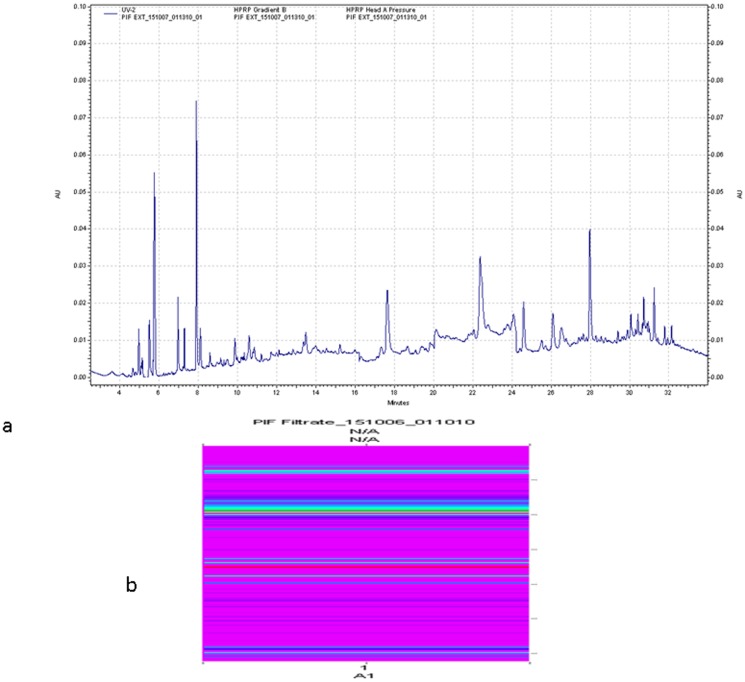
ProteoVue image of PIF-affinity chromatography filtrate. **a**) UV chromatogram trace. **b**) The colored image map indicates the regions of most intense protein concentration.

The data generated from the lysate microarrays detailed above demonstrated that PIF potentially targets several proteins in 10-day old mouse embryos. Those experiments did not however provide direct evidence to determine to which, among the proteins identified within a fraction, PIF binds. To obtain this necessary information, an alternative strategy was specifically designed. A PIF-based resin for affinity chromatography was prepared where mouse embryos can be extracted directly with PIF serving as bait to recover only those proteins in (d10) embryos that have significant interaction with PIF. The affinity extracted proteins were analyzed by reversed phase HPLC and the profile generated using the ProteoVue imaging software (Eprogen) is shown (**Fig. 2a,b**). A second extraction was performed which contained a two-fold higher concentration of the embryo extract than the first run. This allowed detection of a larger number of proteins and it also enabled us to determine their relative abundance within the extract using LC/MS/MS. High degree of concordance between the two different extractions was observed, confirming the validity of our simple but robust affinity extraction method for isolating and identifying PIF-protein binding partners. The negative control column is shown. (**Figure S5**).

Remarkably, as seen in **Table 4** **and Table S4 in File S1**, we also found a high degree of concordance of the proteins separated by affinity chromatography compared with those detected in the Biotin-PIF positive embryo microarray analysis. At least 10 identical proteins and several additional similar proteins were found when the two detection methods were compared. The major groups PDI which contains the TRX enzyme and HSP groups were confirmed. This is relevant since embryo extracts as well the LC/MS/MS-based separation method used were different. This simplified affinity PIF-resin method of protein extraction followed by LC/MS/MS detection clearly demonstrates that PIF not only targets a limited number of proteins but that those are highly specific and relevant for embryo well-being. Thus PDI, and HSPs are the PIF major protein interacting groups.

**Table 4:** PIF-affinity chromatography identified PIF targets.

			No. of Unique Peptides	
Identified Proteins - PIF Resin Extraction	Accession	MW	Ext # 1	Ext # 2
**OXIDATIVE STRESS & PROTEIN MISFOLDING**				
Protein disulfide-isomerase	IPI00133522	57 kDa	6	14
protein disulfide-isomerase A4	IPI00271951	72 kDa	4	4
protein disulfide-isomerase A6-like	IPI00986015	47 kDa	3	6
Calreticulin (PDI partner)	IPI00123639	48 kDa	2	5
**HEAT SHOCK & RELATED PROTEINS**				
HSP 90-beta	IPI00554929	83 kDa	10	12
**HSP70 Prot5**	**IPI00319992**	**72 kDa**	**12**	**18**
**Heat shock cognate 71 kDa protein**	**IPI00323357**	**71 kDa**	**1**	**8**
**HSP 90-alpha**	**IPI00330804**	**85 kDa**	**2**	**7**
Transitional endoplasmic reticulum ATPase	IPI00622235	89 kDa	1	8
**14-3-3 protein gamma (coexpressed HSP B6)**	**IPI00230707**	**28 kDa**	**1**	**4**
14-3-3 protein zeta/delta	IPI00116498	28 kDa	2	3
14-3-3 protein epsilon	IPI00118384	29 kDa	0	3
**NEURAL**				
**Tubulin beta-5 chain**	**IPI00117352**	**50 kDa**	**6**	**8**
Tubulin alpha-1A chain	IPI00110753 (+2)	50 kDa	5	5
**VISCERAL**				
Actin, cytoplasmic 1	IPI00110850 (+1)	42 kDa	6	8
Alpha-actinin-4	IPI00118899	105 kDa	1	3
Actin, alpha cardiac muscle 1	IPI00114593 (+2)	42 kDa	0	3
Isoform 2 of Tropomyosin alpha-1 chain	IPI00227835 (+3)	33 kDa	2	6
Isoform 2 of Tropomyosin beta chain	IPI00874728	33 kDa	0	3
Tropomyosin alpha-4 chain	IPI00421223	28 kDa	2	5

### RIKP amino acid sequence is PIF binding interface with PDI and HSP

PIF was found to bind PDI and HSP, identified as the highest ranking group of proteins. To determine the specific binding site we have used 60 corresponding crystallography derived PDBs obtained from Protein Data Bank (RCSB) using a PepSite 2 server. This enabled PIF binding site identification and corresponding interface residues. Of them 42 were having probability of binding <0.25 and were considered further for binding statistics (**Table 5** **, Table S5 in File S1**). The sequence **M_1_xRIKPxxA_9_** had the highest score, the sequence **RIPK** (arg_3_, ile_4_, lys_5_, pro_6_) was considered as a consensus one.

**Table 5:** PepSite 2 server prediction of PIF residues participating in targets binding site.

	PIF_1-10_ residues and target residues positions									
	M	V	R	I	K	P	G	S	A	N
**N**	29	19	35	27	30	31	10	14	21	19
**at that position [%]**	***69.05***	***45.24***	***83.33***	***64.29***	***71.43***	***73.81***	***23.81***	***33.33***	***50.00***	***45.24***
**out of all occurrences [%]**	***12.34***	***8.09***	***14.89***	***11.49***	***12.77***	***13.19***	***4.26***	***5.96***	***8.94***	***8.09***

Using de novo designed PIF and protein target crystallographic PDB models we first created preliminary manual peptide ligand-protein receptor docking models. In these models PIF was superimposed using the PepSite 2 predicted coordinates of PIF binding. Then the preliminary model was submitted to the currently available flexible peptide docking server - FlexPepDock for refined docking. This docking has a precision of less than 2 Å when compared to native models.

The high precision docking models obtained were further used for *in silico* mutagenesis. We virtually mutated every single amino acid from the entire PIF1-15 sequence participating in the model interface and assessed the change in free energy of the ligand-receptor bond, thus obtaining the amino acids having the highest importance for the binding of PIF to its protein targets. This was achieved using the algorithm implementation of BeaTMusiC server. We statistically evaluated the frequency of residue positions having highest energy impact (**Table 6** **, Table S6 in File S1**). Here again the RIKP sequence had the most frequent employment in the binding interfaces assessed through their *in silico* mutations.

**Table 6:** BeATMuSiC server predicted in silico mutagens disrupting the interface of the PIF docking models with several targets.

	PIF_1-15_ residues and target residues positions														
	M	V	R	I	K	P	G	S	A	N	K	P	S	D	D
**N**	5	3	0	19	3	7	0	0	0	3	0	1	0	0	0
**at that position [%]**	***15***	***9***	***0***	***57***	***9***	***21***	***0***	***0***	***0***	***9***	***0***	***3***	***0***	***0***	***0***
**out of all occurrences [%]**	***12***	***7***	***0***	***46***	***7***	***17***	***0***	***0***	***0***	***7***	***0***	***2***	***0***	***0***	***0***

### *In silico* PDI docking and PIF mutagenesis reveal that Pro_6_ and Val_2_ are PDI conformational dependent mutations important for PIF binding

**Figure 3:**
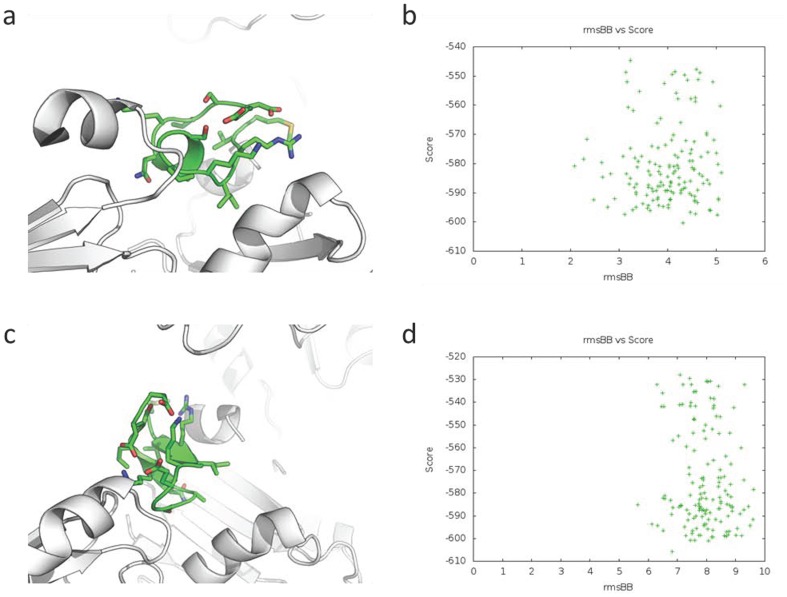
Prove of concept of the *in silico* mutagenesis validation of the flexible peptide docking of PIF to PDI. a) Result of flexible docking of PIF**^wt^** to reduced PDI (PDB 4EKZ) using *de novo* generated PIF model 1 (PepFold) manually pre-aligned to the PepSite 2 predicted PIF^wt^ residues center of mass. PyMOL script generated image by the FlexPepDock server of the new flexibly adjusted to the PDI surface interface PIF peptide model generated by the docking algorithm. Viewing angle is selected automatically. b) FlexPepDock generated distribution of the best score (model total ΔG decrease) vs rmsBB (root square mean of the backbone distance to startRMSD, in this case 0) of the PIFwt models docked at 2 to 3 Å distance from the initial state. c) Result of the flexible re-docking of PIF**^mutant-1^** to reduced PDI (PDB 4EKZ) using *de novo* generated PIF**^mutant-1^** model-1 (PepFold) manually pre-aligned to the PepSite 2 predicted for PIF**^wt^** residues center of mass, and PIF^wt^ docked to PDI refined FlexPepDock model submitted as a reference. PyMOL script generated image by the FlexPepDock server of the new flexibly adjusted to the PDI surface interface PIF**^mutant-1^** peptide model generated by the docking algorithm. Viewing angle is selected automatically. d) FlexPepDock generated distribution of the best score (model total ΔG decrease) vs rmsBB (root square mean of the backbone distance to startRMSD of the PIF**^wt^** to PDI docked model) of the PIF**^mutant-1^** models docked at 6–8 Å distance from the PIF**^wt^** state.

**Figure 4:**
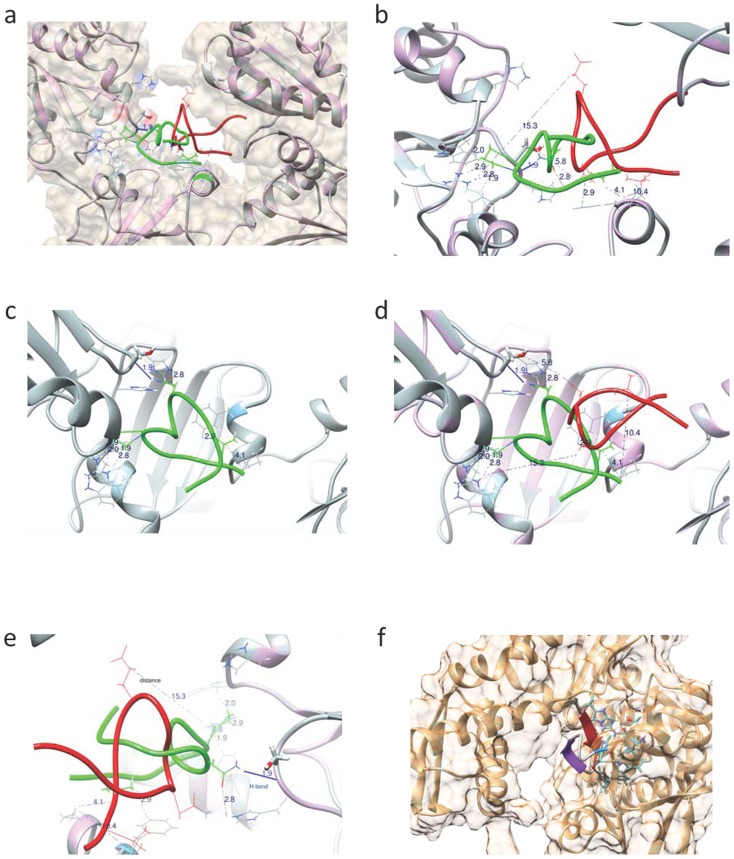
PIF to PDI binding interface *in silico* analysis. HSP70 predicted binding by PIF. a) Super-position (Image generated by UCSF Chimera) of PIF**^mutant-1^_1-15_** docking model (PIF, red; PDI, purple) over PIF**^wt^_1-15_** docking model (PIF green; PDI, grey), both generated by FlexPepDock. PDI molecular surface is transparent. b–e) Close views of the PIF^wt^ binding interface to the PDI. PIF residues Val_2_, Pro_6_ and Asn_10_ and multiple PDI residues are wire-framed and colored as the principal molecules. b) Side view. c) Top view of PIF^wt^ binding alone. d) Top view of PIF^wt^ and PIF^mutant-1^ binding. Three separate coiled coils form close proximity weak interactions (distances between 1.9–4.1 Å) with native PIF molecule (green), and one H-bond between PDI and Asn_10_ (1.9 Å). Mutated PIF (in red) “loses” at least two of these weak interactions and the H-bond. e) Rear view showing that mutated PIF have increased distance of Val_2_ and Pro_6_ to the PDI interface of 10 and 15 Å accordingly. f) Model of PIF**^wt^_1-15_** docked to HSP70 (PDB 3FZH) as predicted by PepSite 2 (p = 0.1195) rendered by Chimera. A *de novo* generated PIF^wt^ model 6 (PepFold) manually pre-aligned to the PepSite 2 predicted PIF^wt^ residues center of mass over HSP70 crystallography derived model. Model 6 has one beta sheet and was selected as maximally overlapping the PepSite 2 predicted residue positions. HSP70 interface residues are blue and red heteroatom colored and rendered as ball and stick style.

Using *in silico* mutagenesis (BeaTMusiC) of refined flexible docking models (FlexPepDock) we found two conformation dependent and one conformation independent PIF mutations (**Table S7 in File S1**). Namely Ile_4_ is conformation independent, as it was present in both reduced and oxidized state of PDI, while the two mutations: Pro_6_ - specific to the reduced form, and Val_2_ - specific to the oxidized form are conformation dependent. Comparing PIF secondary structure mutation wise, wtPIF_1-15_ had 5 beta turns and 3 gamma turns (PDBsum, EMBL-EBI), and the RIKP sequence participated in a gamma turn (Arg_3_-Lys_5_) and in a beta turn (Lys_5_-Ser_8_). The conformation-independent mutants (Ile_4_: mut2 and mut3) have either 1 beta sheet, 1 beta hairpin, 3 beta bulges, 2 strands and 1 beta turn or 4 beta turns and 2 gamma turns accordingly. The PDI reduced conformation dependent mutant (Pro_6_: mut1) on the other hand had 1 beta sheet, 1 beta hairpin, 3 beta bulges, 2 strands and 1 beta turn, while the oxidative state specific mutant (Val_2_: mut4) has only 2 beta turns. These structural changes suggest that mutations induce altered PIF peptide fluctuability and reduced bonding at its interface. The *in silico* mutagenesis demonstrated an increased distance of the PIF models assessed by the backbone distance between the mutated and the referent wt PIF model towards the PDI protein target (**Figure 3a, b**), thus supporting the hypothesis of the particular residue importance generated using BeaTMusic (**Figure 3c,d**). (**Figure 4 a–e**). The energy of *in silico* flexible docking of wt PIF and mutant PIF models are described in (**Table 7**).

**Table 7:** Energy of *in silico* flexible docking of wt PIF and mutant PIF models to crystallography derived model of PDI.

Docking Scenario	PDB	Score	I score [kkal/mol]	pep score [kkal/mol]	rmsBB [Å]	rmsBB interface [Å]	start RMSbb
**PDI oxidized+wt PIF**	4EKZ	−598.016	−18.034	−13.601	5.281	4.525	0
**PDI oxidized+mutant 1 PIF**	4EKZ	−605.118	−16.79	−17.604	16.012	15.135	6.723
**PDI oxidized+mutant 2 PIF**	4EKZ	−600.675	−14.534	−18.53	10.432	10.058	6.723
**PDI reduced, sub. A+wt PIF**	4ELI.A	−581.9	−16.776	−14.119	5.389	5.618	0
**PDI reduced, sub. B+wt PIF**	4ELI.B	−637.393	−10.11	−9.5	6.855	6.525	0

**Score** - **Total Rosetta energy** for this model,

**I score** - **Interface energy** (The total energy of the complex less the energy of the partners when separated).

**pep score** - The sum of energies contributed only by peptide residues.

**rmsBB** - RMSD is calculated only for peptide backbone heavy atoms.

**rmsBB interface** - RMSD is calculated only for heavy backbone atoms of peptides residues at the interface.

### HSP70 is additional PIF target demonstrated by PepSite prediction

Similarly, crystal structures of HSP70 (models 3FZH, 3A8Y) were assessed for PIF putative docking sites by PepSite 2 (**Figure 4f**). This confirmed that PIF interacts with the RIPK in HSP70 protein. This revealed that HSP70 bound to Bag1 (models 3FZH) or to BAG5 BD5 (models 3A8Y) become PIF targets. Analysis of HSP interaction with small molecules is shown. (**Figure S6**) This data imply that BCL2-associated athanogens enhance the anti-apoptotic effects of BCL2, and could be another target of PIF.

## Discussion

Herein, we show that PIF directly and specifically targets viable cultured embryos. Further we demonstrate by using two complementary methods, that PIF targets principally oxidative stress, and protein misfolding-regulating proteins, PDI and HSPs, sharing essential RIPK binding site. PIF also targets promiscuous tubulins and actins involved in neural and somatic development. Data reveal PIF targets known to play a critical role in embryogenesis.

PIF's beneficial effect on embryo development and protection has been already demonstrated in culture. PIF secreted by viable embryos has an autotrophic effect which is blocked by using an anti-PIF monoclonal antibody [19], [22]. PIF also added to singly cultured embryos promotes their development. Significantly embryo toxic serum effect is negated by addition of PIF to the culture [22]. Therefore it was of great importance to examine which are the PIF-specific targets that lead to this protective effect. We confirmed by affinity chromatography that PIF targets critical proteins Involved in regulating oxidative stress and protein misfolding: Generating such data provides an important facet in understanding global PIF-embryo relationship. The highest ranking protein group identified from MS analysis following affinity chromatography extraction is protein disulfide-isomerases (PDI). This enzyme is present in the endoplasmic reticulum and is a thiol oxidoreductase family member. This class of enzyme catalyzes formation and isomerization of intra- and inter-chain disulfide bridges. Moreover, PDI is also the β-subunit of prolyl-4-hydroxylase (PH4) which hydroxylates proline residues in Xaa-Pro-Gly sequences on single collagen polypeptide chains [39]. The PDI enzyme has two TRX motifs and plays a major role in oxidative stress control as well as prevention of protein misfolding. Beyond PDI, two additional PDI family members were identified from the affinity extracts; PDIA4, and PDIA6-like, both having a number of active TRX motifs. The PDIA4 is also a regulator of insulin degradation and the PDI and PDIA4 have highly similar catalytic domains.

The other oxido-reductive enzyme also found in a biotin-PIF positive microarray fraction, PDRX4, is reduced by PDI demonstrating that oxidation by H_2_O_2_ can be coupled with disulfide formation [40]. Both the placenta and post-implantation embryos express TRX which is amplified at the aerobic stage of development reflecting critical need for oxygen control [41], [42]. This result indicates that PIF binding to proteins related to oxidative stress is a significant mechanism of PIF-embryo targeting.

PIF targets HSP, as confirmed by affinity chromatography. A second major group of proteins, HSPs, were also identified and confirmed by analyzing HPLC fractions/microarray analysis. A number of proteins match exactly those identified in the Biotin-PIF positive fractions. The HSPs are conserved molecular chaperones which control signal transduction, protein folding, degradation and morphogenesis. These critical proteins complement PDI-related proteins in protecting the embryo against an adverse environment since anti-HSP70 antibody added in culture impairs embryo development [43].

The *in vivo* juvenile diabetes model documents PIF's regulatory role on both PDI and HSP. Long-term protection against diabetes led to preserved islet architecture and insulin expression. Proteomic analysis of the pancreas demonstrated that the ranking proteins which were up-regulated are the PDI and HSP family members. Further, both the neuroinflammation and GVHD models demonstrated that the highest ranking pathway/mechanism of PIF-induced protection was against oxidative stress and protein misfolding [27], [28], [30], [31], [44] [45], [46] [47]. Thus fundamental mechanisms through which PIF operates in the embryo are clearly translatable for non-pregnant immune disorder models. Further, due to PIF's high safety profile in FDA-mandated toxicology studies, a fast track designation was granted by the FDA to initiate clinical trials for the treatment of immune disorders.

RIPK is the consensus binding site for PIF to PDI and HSP proteins. The top two ranking PIF interacting groups contain several similar members. The *in silico* analysis has enabled identification of the PIF binding site for PDI as well as HSP. Four complementary methods of analysis were used for determining PIF-interacting site. Remarkably, despite the presence of major sequence and structural differences between members of the two groups analysis revealed that PIF targets a similar amino acid sequence-RIPK in both. Further modification of a single amino acid in the PIF structure led to major deformation of the helical structure of the peptide with increased distance and energy required for effective interaction. Such data provides evidence for site specific interaction of PIF with its major targets.

PIF targets highly flexible promiscuous tubulins and actins: Furthermore, PIF interaction with tubulins, actins, and Eef1a1 was confirmed. These proteins play an important role in both neural and vascular development. Conversely, their altered expression or mutation adversely affects embryos. Specific binding of PIF to actins is expected since they are highly polymorphic and interact with >150 partners [48]. Actin folding also requires a chaperone like HSP90, which is also a major PIF target. Thus both structurally and functionally PIF enables multi-targeted interaction.

Results also show that methods used for identifying potential PIF-interacting proteins are robust and reproducible. Therefore, single step isolation used enables a comprehensive evaluation of peptide- proteins interaction in high fidelity.

**Figure 5:**
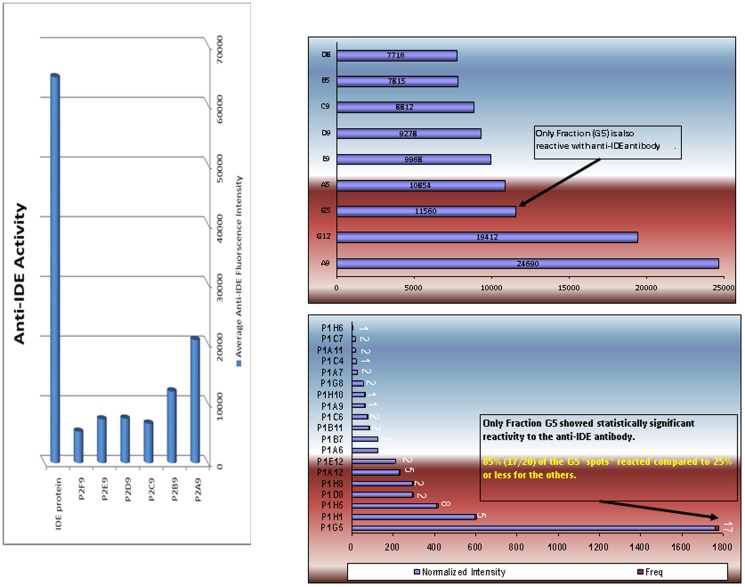
Biotin-PIF binds significantly to Anti-IDE positive protein factions. **a**) Averaged normalized intensity data is represented for 20 separate analyses. **b**) Among the tested fractions, only G5 was identified by the Anti-IDE antibody. **c**) Only fraction G5 shows statistically significant reactivity with antibody. P<0.003. The frequency of the fraction showing positive reactivity is indicated above the intensity bar.

PIF targets insulin degrading enzyme (IDE). In Protoarray analysis PIF was shown to bind to a limited number of intracellular targets, the most pronounced among them being insulin degrading enzyme (IDE) [23]. Since both analytical methods failed to identify this protein using LC/MS/MS, we therefore have used immunochemistry, a more sensitive method to probe those Biotin-PIF positive fractions. We found that the anti-IDE antibody binds avidly only to the A9 fraction and to a lesser degree to the G5 and F6 protein fractions, as measured by fluorescence intensity. In contrast, in the 93 other fractions no significant binding was noted. To document that the binding is specific, the experiment was repeated several times on a number of arrays confirming the validity of these observations. Furthermore, purified IDE was run in parallel to document that the binding to IDE is replicated using a side by side testing (**Fig. 5a,b,c**). In addition, we demonstrate that the binding was statistically significant as replicated over 85% (17/20) of the G5 spots tested with anti-IDE-antibody as compared with control 25% (5/20), P = 0.0003. The third fraction where IDE activity was identified was F6 where the binding of the antibody was moderate. This indicates that PIF directly targets the embryo and the site of binding is likely to be IDE.

**Figure 6:**
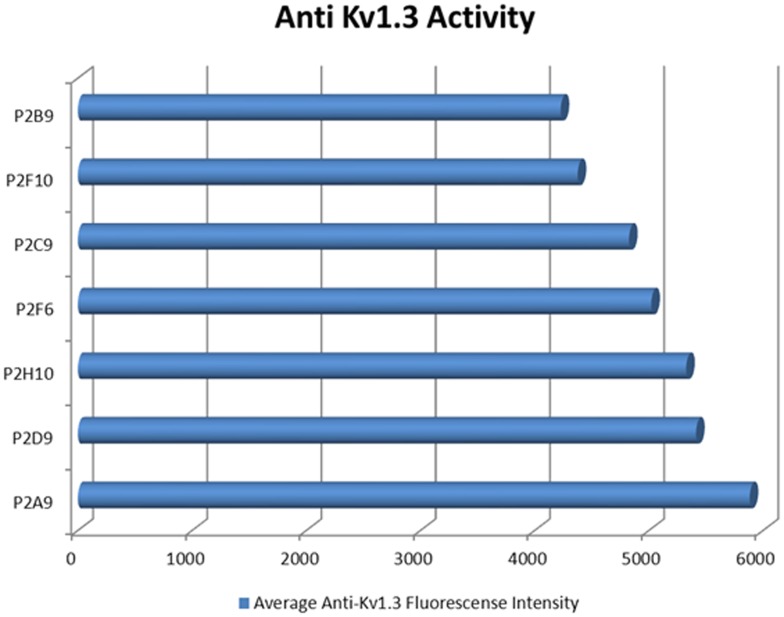
Biotin-PIF binds significantly to anti-KV1.3b positive fractions. Averaged normalized intensity data is represented. The highest intensity was found in the A9 fraction. The frequency of the fraction showing positive reactivity is indicated above the intensity bar.

PIF targets Kv1.3-beta channel which is the binding site of cortisol. ProtoArray analysis demonstrated that PIF also interacts with two different variants of the potassium channel Kv1.3 beta [23]. This channel controls the alpha pore and is the binding site of cortisol. When examined by using LC/MS/MS however, no match for this protein was identified. Therefore, we tested whether this channel is also present in the PIF positive fractions using an anti-Kv1.3 purified polyclonal antibody tested at different dilutions. The A9 fraction (**Fig. 6**) showed moderate binding while in other fractions binding was limited. This implies that the concentration of this protein is probably very low or is below the threshold of detection by the LC/MS/MS method. Nonetheless we were able to detect the channel by using the immunohistochemistry-based sensitive method.

PIF is an embryo specific peptide secreted only by viable embryos. It is detected in maternal circulation around the time of implantation leading to a favorable pregnancy outcome. The endogenous regulatory effect of PIF on the embryo enables protection against maternal adversity- since the embryo's own immune system becomes functional later [22], [26]. The current study provides support for such a premise.

## Conclusions

We document that PIF is up-taken only by viable embryos. Also direct binding of oxidative stress and protein misfolding PDI and HSPs proteins sharing the similar RIPK binding site is identified for the first time. This data provides important insight into endogenous PIF involvement in pathways critical for embryo protection and survival. Furthermore, by targeting flexible multi-targeted proteins tubulins and actins suggests PIF's involvement in neuronal and vascular development.

The current study used advanced and also novel analytical methods to identify potential PIF-targets within the embryo. Delineation of their precise role and relevance for embryo wellbeing remains to be explored in future studies.

## Supporting Information

Figure S1 **Separated embryo extracts retention time (RT) and corresponding well number.** The extracted embryo proteins were separated by 1D fractionation and placed in a 96 well plate. (representative).
(TIF)

Figure S2 **Microarray analysis and Proteovue of 10-Day old mouse embryos 1D fractions with Biotin-PIF reactivity.** **a**) Reactive fractions are sorted from the highest to lowest for the labeled PIF binding. **b**) ProteoVue image of the Full 1D fractionation analysis with the retention time (RT) regions with highest reactivity for the labeled PIF indicated. The UV chromatogram trace is indicated on the left and the colored image map on the right indicating the regions of most intense protein concentration.
(TIF)

Figure S3 **Proteovue of G5 H5 G12 1D fractions for Biotin-PIF.** Data shows fluorescence intensities for microarray spots reactive to Biotin-PIF. The UV chromatogram trace is indicated on the left and the colored image map on the right indicating the regions of most intense protein concentration.
(TIF)

Figure S4 **ProteoVue image of A9 1D fractionation analysis.** Data shows fluorescence intensities for microarray spots that are reactive to Biotin-PIF. The UV chromatogram trace is indicated on the left and the colored image map on the right indicating the regions of most intense protein concentration.
(TIF)

Figure S5 **Proteovue image of Biotin PIF full fractionation analysis as it is compared to Biotin alone targets (control).** No bands were associated with the control image. The UV chromatogram trace is indicated on the left and the colored image map on the right indicating the regions of most intense protein concentration.
(TIF)

Figure S6 **HSP targeted by PIF.** Crystal Structures of Hsc70/Bag1 and 3A8Y in Complex with Small Molecule Inhibitors PDB: 3FZH. Using the PepSite Server, the significance of association was determined.
(TIF)

File S1 **Table S1.** Biotin-PIF binds the G12 fraction in mouse embryo extracts. **Table S2.** Biotin-PIF binds the B9 fraction in mouse embryo extracts. **Table S3.** Biotin-PIF binds the A9 fraction in mouse embryo extracts. **Table S4.** (additional proteins identified from Table 4). **Table S5.** PepSite 2 prediction of PIF residues participating in targets binding site. **Table S6.** BeATMuSiC server predicted *in silico* mutagens disrupting the interface of the PIF docking models with several targets. **Table S7.** PIF mutant models.
(DOCX)
