# Correction: Insight into PreImplantation Factor (PIF*) Mechanism for Embryo Protection and Development: Target Oxidative Stress and Protein Misfolding (PDI and HSP) through Essential RIPK Binding Site

**DOI:** 10.1371/journal.pone.0113886

**Published:** 2014-11-12

**Authors:** The PLOS ONE Staff

An error appears throughout the published article. RIPK should appear as RIKP.

## References

[1] Barnea ER, Lubman DM, Liu Y-H, Absalon-Medina V, Hayrabedyan S, et al (2014) Insight into PreImplantation Factor (PIF*) Mechanism for Embryo Protection and Development: Target Oxidative Stress and Protein Misfolding (PDI and HSP) through Essential RIPK Binding Site. PLoS ONE 9(7): e100263 doi:10.1371/journal.pone.0100263 24983882 10.1371/journal.pone.0100263 PMC4077574

