# Supplementary figures and images for: Insight into PreImplantation Factor (PIF*) Mechanism for Embryo Protection and Development: Target Oxidative Stress and Protein Misfolding (PDI and HSP) through Essential RIPK Binding Site
Source: PLoS One. 2014 Jul 1;9(7):e100263. doi: 10.1371/journal.pone.0100263 (PMC4077574; doi:10.1371/journal.pone.0100263)

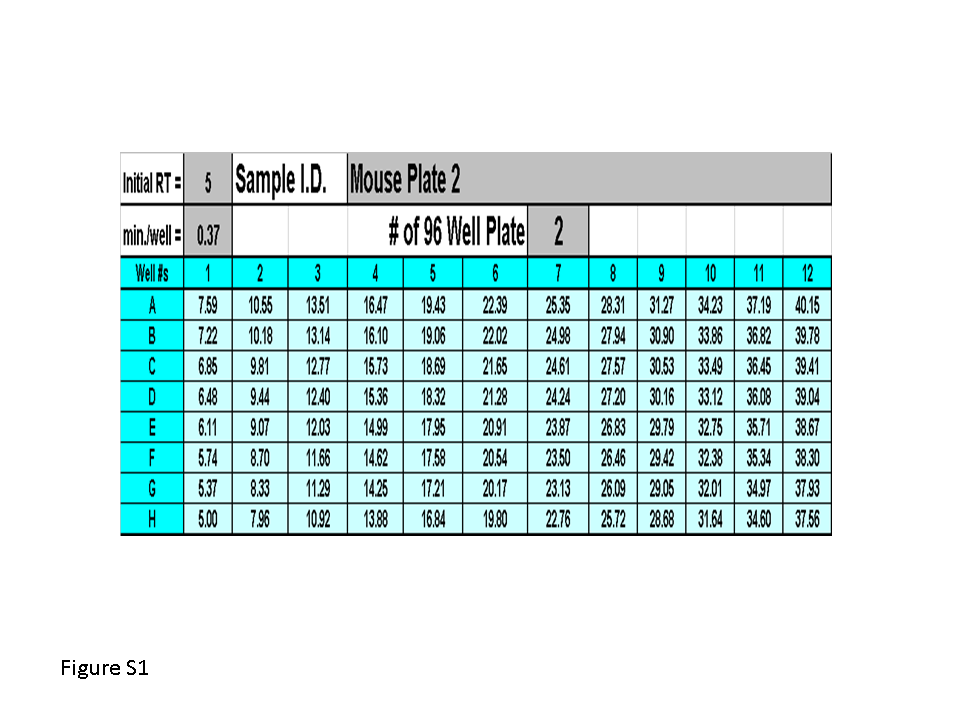

Supplement: Figure S1 — Separated embryo extracts retention time (RT) and corresponding well number. The extracted embryo proteins were separated by 1D fractionation and placed in a 96 well plate. (representative). (TIF) [file pone.0100263.s001.tif]

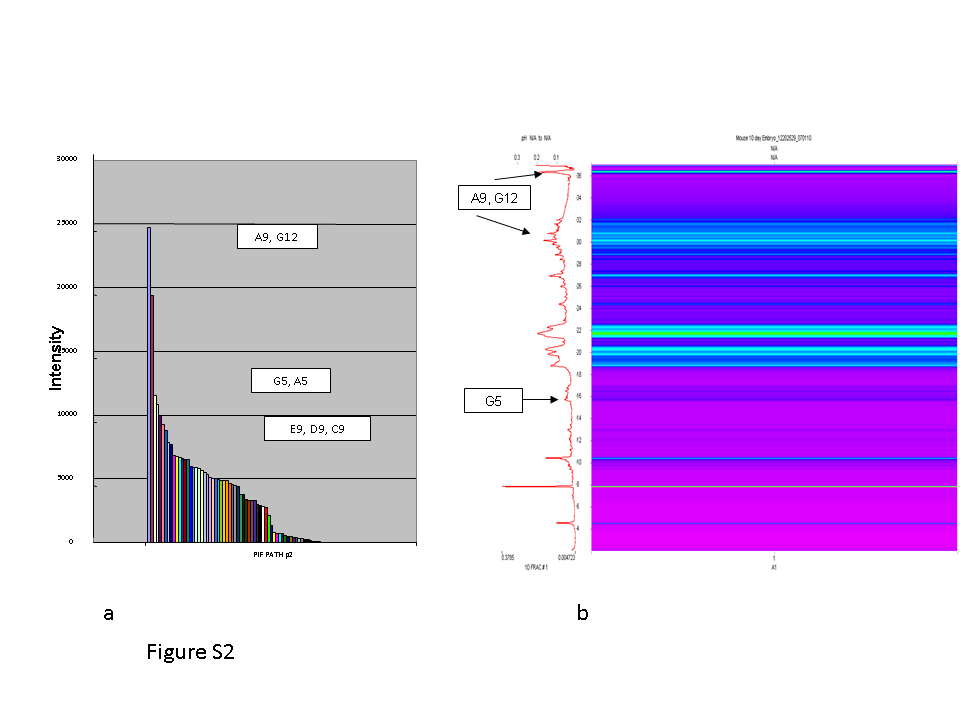

Supplement: Figure S2 — Microarray analysis and Proteovue of 10-Day old mouse embryos 1D fractions with Biotin-PIF reactivity. a) Reactive fractions are sorted from the highest to lowest for the labeled PIF binding. b) ProteoVue image of the Full 1D fractionation analysis with the retention time (RT) regions with highest reactivity for the labeled PIF indicated. The UV chromatogram trace is indicated on the left and the colored image map on the right indicating the regions of most intense protein concentration. (TIF) [file pone.0100263.s002.tif]

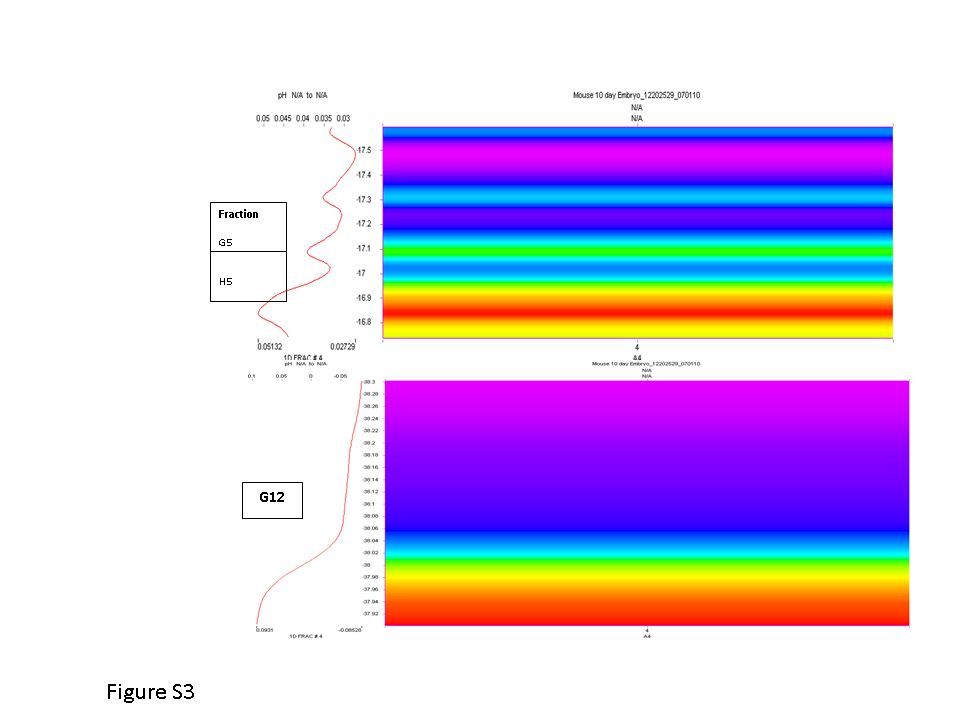

Supplement: Figure S3 — Proteovue of G5 H5 G12 1D fractions for Biotin-PIF. Data shows fluorescence intensities for microarray spots reactive to Biotin-PIF. The UV chromatogram trace is indicated on the left and the colored image map on the right indicating the regions of most intense protein concentration. (TIF) [file pone.0100263.s003.tif]

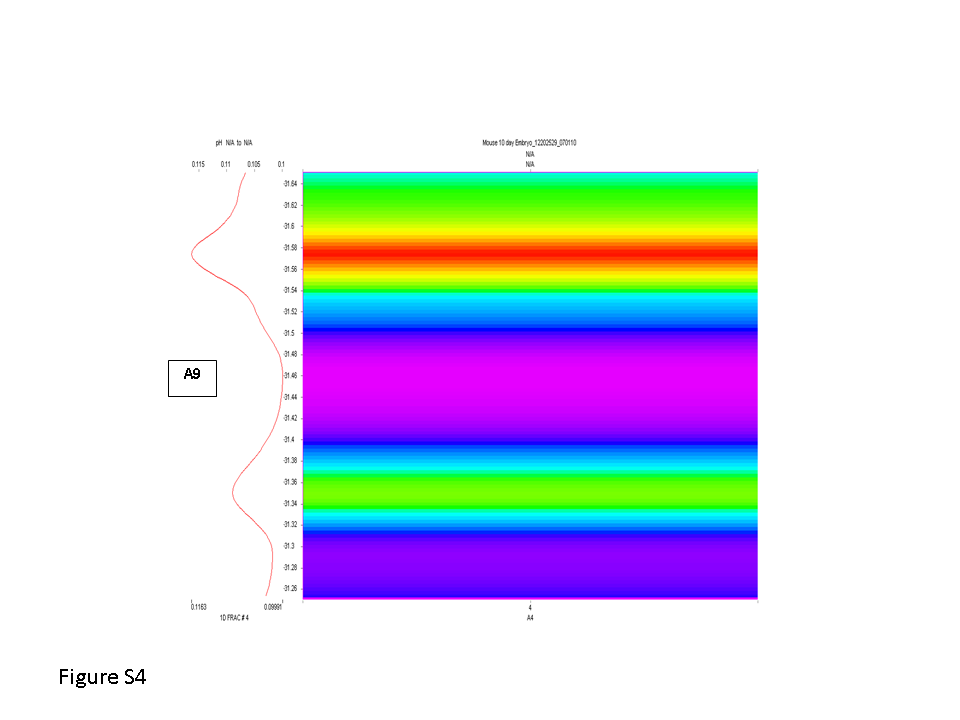

Supplement: Figure S4 — ProteoVue image of A9 1D fractionation analysis. Data shows fluorescence intensities for microarray spots that are reactive to Biotin-PIF. The UV chromatogram trace is indicated on the left and the colored image map on the right indicating the regions of most intense protein concentration. (TIF) [file pone.0100263.s004.tif]

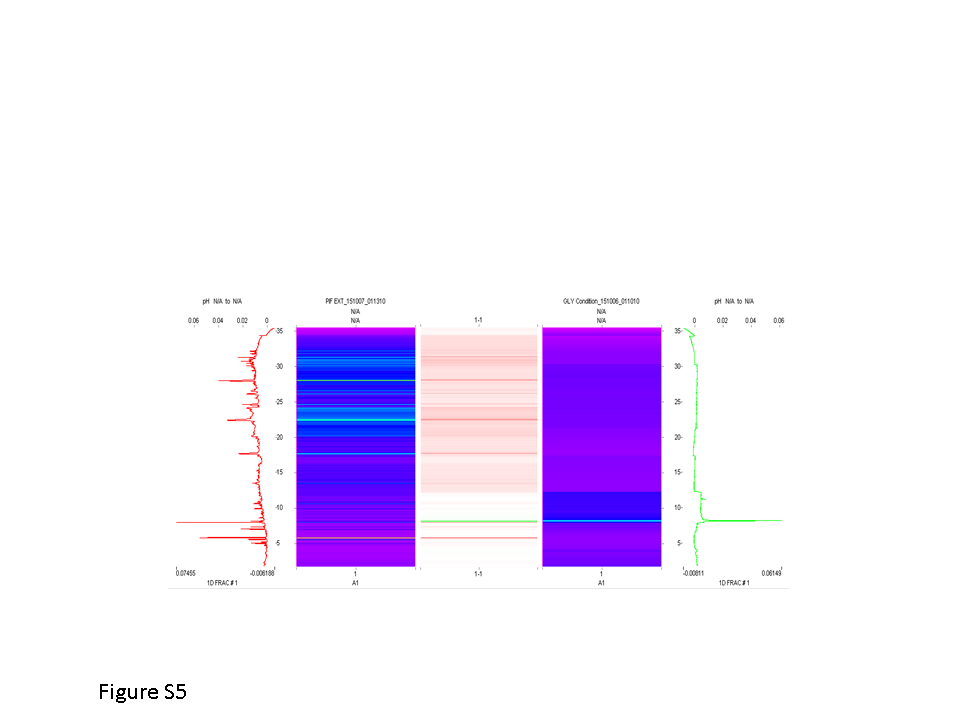

Supplement: Figure S5 — Proteovue image of Biotin PIF full fractionation analysis as it is compared to Biotin alone targets (control). No bands were associated with the control image. The UV chromatogram trace is indicated on the left and the colored image map on the right indicating the regions of most intense protein concentration. (TIF) [file pone.0100263.s005.tif]

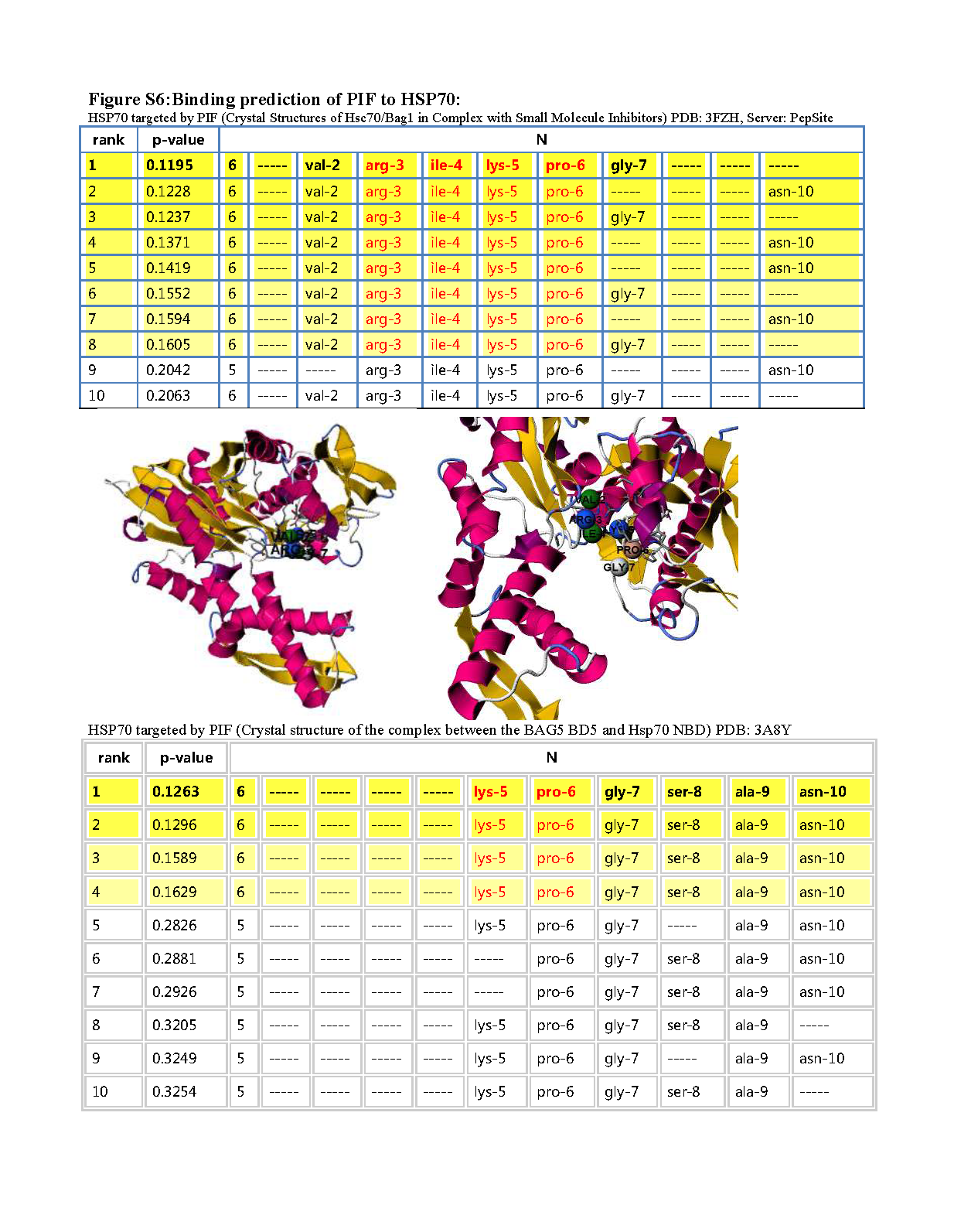

Supplement: Figure S6 — HSP targeted by PIF. Crystal Structures of Hsc70/Bag1 and 3A8Y in Complex with Small Molecule Inhibitors PDB: 3FZH. Using the PepSite Server, the significance of association was determined. (TIF) [file pone.0100263.s006.tif]
