# Supplementary material for: Insight into PreImplantation Factor (PIF*) Mechanism for Embryo Protection and Development: Target Oxidative Stress and Protein Misfolding (PDI and HSP) through Essential RIPK Binding Site
Source: PLoS One. 2014 Jul 1;9(7):e100263. doi: 10.1371/journal.pone.0100263 (PMC4077574; doi:10.1371/journal.pone.0100263)
Supplement: File S1 — Table S1. Biotin-PIF binds the G12 fraction in mouse embryo extracts. Table S2. Biotin-PIF binds the B9 fraction in mouse embryo extracts. Table S3. Biotin-PIF binds the A9 fraction in mouse embryo extracts. Table S4. (additional proteins identified from Table 4). Table S5. PepSite 2 prediction of PIF residues participating in targets binding site. Table S6. BeATMuSiC server predicted in silico mutagens disrupting the interface of the PIF docking models with several targets. Table S7. PIF mutant models. (DOCX) [file pone.0100263.s007.docx]

Table S1 – Biotin-PIF binds the G12 fraction in mouse embryo extracts

| **Description** | **Accession** | **Coverage** | **# Peptides** | **# AAs** | **MW [kDa]** | **calc. pI** | **Score** |
| --- | --- | --- | --- | --- | --- | --- | --- |
| HBS1-iso 3 | IPI:IPI00275176.3 | 3.10 | 14 | 612 | 67.5 | 5.64 | 7.32 |
| HBS1-iso2 | IPI:IPI00464282.4 | 2.84 | 11 | 599 | 65.2 | 8.34 | 7.32 |
| PRDX4 | IPI:IPI00108426.2 | 16.96 | 10 | 112 | 12.7 | 10.27 | 7.00 |
| DENN/MADD domain 5B | IPI:IPI00889876.2 | 0.67 | 3 | 1042 | 118.7 | 6.55 | 4.97 |
| Actin-related protein 6 | IPI:IPI00110922.2 | 3.28 | 4 | 396 | 45.8 | 5.03 | 4.77 |
| Iso1 Activating signal coin1 | IPI:IPI00135240.1 | 4.48 | 27 | 581 | 66.2 | 7.55 | 4.73 |
| Iso 2 Centlein | IPI:IPI00621351.1 | 2.20 | 3 | 318 | 35.7 | 8.48 | 4.52 |
| Cytochrome P450 26A1 | IPI:IPI00115829.1 | 1.41 | 3 | 497 | 56.1 | 8.60 | 4.49 |
| scleraxis | IPI:IPI00381245.2 | 5.80 | 3 | 207 | 22.2 | 10.14 | 3.71 |
| dynein, axon, heavy11 | IPI:IPI00622122.5 | 0.80 | 9 | 4488 | 516.0 | 6.16 | 1.72 |
| Iso 1 of Nischarin | IPI:IPI00110435.2 | 1.51 | 12 | 1593 | 174.9 | 5.16 | 1.61 |
| RAB12 | IPI:IPI00169699.4 | 2.41 | 1 | 291 | 32.2 | 8.84 | 1.61 |
| Iso1Phostensin | IPI:IPI00607928.1 | 3.03 | 4 | 594 | 65.6 | 5.39 | 1.54 |
| DDB1/ CuL4- factor 6 | IPI:IPI00120084.1 | 2.40 | 3 | 876 | 97.5 | 5.22 | 0.00 |
| Cadherin EGF LAG 7-GR 1 | IPI:IPI00127701.1 | 0.73 | 3 | 3034 | 330.3 | 5.91 | 0.00 |
| Iso 2Glutamate decarbo- 1 | IPI:IPI00316617.8 | 4.37 | 4 | 526 | 59.8 | 7.25 | 0.00 |
| Gm10414 | IPI:IPI00654290.1 | 6.11 | 1 | 131 | 14.0 | 10.62 | 0.00 |
| collagen,XXII, a 1 | IPI:IPI00944073.1 | 1.61 | 6 | 1613 | 159.8 | 7.21 | 0.00 |

Table S2 Biotin-PIF binds the B9 fraction in mouse embryo extracts.

| **Description** | **Accession** | **Coverage** | **# Peptides** | **# AAs** | **MW [kDa]** | **calc. pI** | **Score** |
| --- | --- | --- | --- | --- | --- | --- | --- |
| Hb Y embryonic | IPI:IPI00133148.2 | 30.61 | 10 | 147 | 16.1 | 8.19 | 75.19 |
| 14-3-3 protein gamma | IPI:IPI00230707.6 | 17.41 | 14 | 247 | 28.3 | 4.89 | 104.91 |
| 14-3-3 protein sigma | IPI:IPI00118286.1 | 13.31 | 9 | 248 | 27.7 | 4.83 | 62.26 |
| 78 kDa glucose-regulated protein | IPI:IPI00319992.1 | 14.81 | 15 | 655 | 72.4 | 5.16 | 78.17 |
| Actin, alpha skeletal muscle | IPI:IPI00110827.1 | 19.10 | 41 | 377 | 42.0 | 5.39 | 261.66 |
| ADP-ribosylation factor 1 | IPI:IPI00221613.5 | 27.62 | 12 | 181 | 20.7 | 6.80 | 61.57 |
| Alpha-actinin-1 | IPI:IPI00380436.1 | 7.51 | 10 | 892 | 103.0 | 5.38 | 58.37 |
| Annexin A2 | IPI:IPI00468203.3 | 15.63 | 11 | 339 | 38.7 | 7.69 | 63.68 |
| ATP synthase subunit beta, mitochondrial | IPI:IPI00468481.2 | 27.03 | 33 | 529 | 56.3 | 5.34 | 167.15 |
| Beta-actin-like protein 2 | IPI:IPI00221528.1 | 11.97 | 19 | 376 | 42.0 | 5.49 | 131.32 |
| Cholesterol side-chain cleavage enzyme, mitochondrial | IPI:IPI00136928.3 | 12.74 | 10 | 526 | 60.3 | 9.39 | 56.56 |
| Cytoskeleton-associated protein 4 | IPI:IPI00223047.2 | 14.96 | 13 | 575 | 63.7 | 5.64 | 89.56 |
| D-3-phosphoglycerate dehydrogenase | IPI:IPI00225961.5 | 11.63 | 9 | 533 | 56.5 | 6.54 | 46.88 |
| Dolichyl-diphosphooligosaccharide-glycosyltransferase 1 | IPI:IPI00309035.2 | 13.82 | 13 | 608 | 68.5 | 6.46 | 83.05 |
| Elongation factor 1-alpha 1 | IPI:IPI00307837.6 | 10.82 | 11 | 462 | 50.1 | 9.01 | 55.06 |
| Elongation factor 2 | IPI:IPI00466069.3 | 6.76 | 12 | 858 | 95.3 | 6.83 | 101.49 |
| Endoplasmin | IPI:IPI00129526.1 | 8.10 | 15 | 802 | 92.4 | 4.82 | 85.31 |
| Eukaryotic initiation factor 4A-I | IPI:IPI00118676.3 | 19.21 | 19 | 406 | 46.1 | 5.48 | 108.38 |
| Glucose-6-phosphate isomerase | IPI:IPI00669556.2 | 12.50 | 11 | 344 | 38.3 | 8.48 | 60.58 |
| Heat shock cognate 71 kDa protein | IPI:IPI00323357.3 | 27.24 | 38 | 646 | 70.8 | 5.52 | 191.68 |
| Heat shock protein HSP 90-alpha | IPI:IPI00330804.4 | 23.74 | 57 | 733 | 84.7 | 5.01 | 335.51 |
| Inositol-3-phosphate synthase 1 | IPI:IPI00119886.1 | 6.10 | 12 | 557 | 60.9 | 6.42 | 59.49 |
| Isoform 1 of 60 kDa heat shock protein, mitochondrial | IPI:IPI00308885.6 | 10.12 | 12 | 573 | 60.9 | 6.18 | 63.27 |
| IsoL Complement C3 (Fragment) | IPI:IPI00323624.3 | 11.00 | 26 | 1663 | 186.4 | 6.81 | 215.82 |
| L-lactate dehydrogenase A chain | IPI:IPI00319994.6 | 17.47 | 9 | 332 | 36.5 | 7.74 | 143.66 |
| Myosin-9 | IPI:IPI00123181.4 | 11.43 | 29 | 1960 | 226.2 | 5.66 | 200.11 |
| Nicotinamide Pribosyltransferase | IPI:IPI00320188.5 | 14.66 | 9 | 491 | 55.4 | 7.15 | 50.16 |
| Perilipin-3 | IPI:IPI00319270.2 | 19.91 | 13 | 437 | 47.2 | 5.62 | 60.60 |
| Plastin-2 | IPI:IPI00118892.6 | 18.82 | 29 | 627 | 70.1 | 5.33 | 175.17 |
| Profilin actin binder | IPI:IPI00650039.1 | 39.29 | 10 | 112 | 11.8 | 4.88 | 46.89 |
| Putative uncharacterized protein | IPI:IPI00473320.2 | 17.65 | 41 | 374 | 41.8 | 6.30 | 264.45 |
| Atp5a1 | IPI:IPI00857439.1 | 16.50 | 11 | 503 | 54.6 | 8.24 | 78.49 |
| Ribonucleoside-diP reductase M2 | IPI:IPI00112645.1 | 16.67 | 10 | 390 | 45.1 | 5.45 | 48.67 |
| Serine protease inhibitor A3K | IPI:IPI00131830.1 | 16.51 | 14 | 418 | 46.8 | 5.16 | 74.91 |
| similar to Eef 4AI isoform 1 | IPI:IPI00462110.2 | 10.90 | 10 | 376 | 42.7 | 5.29 | 62.50 |
| similar (GAPDH) 3isoform 2 | IPI:IPI00850243.1 | 13.89 | 10 | 288 | 30.8 | 7.75 | 53.84 |
| T-complex protein 1 subunit beta | IPI:IPI00320217.9 | 17.20 | 15 | 535 | 57.4 | 6.40 | 80.58 |
| Tubulin alpha-1C chain | IPI:IPI00403810.2 | 12.92 | 11 | 449 | 49.9 | 5.10 | 89.40 |
| Tubulin beta-2A chain | IPI:IPI00338039.1 | 24.27 | 25 | 445 | 49.9 | 4.89 | 145.11 |
| Tubulin beta-2C chain | IPI:IPI00169463.1 | 28.99 | 27 | 445 | 49.8 | 4.89 | 163.10 |
| Tubulin beta-4 chain | IPI:IPI00109073.5 | 19.59 | 17 | 444 | 49.6 | 4.88 | 106.20 |
| Tubulin beta-5 chain | IPI:IPI00117352.1 | 28.60 | 34 | 444 | 49.6 | 4.89 | 183.97 |
| Tubulin beta-6 chain | IPI:IPI00122928.1 | 15.88 | 16 | 447 | 50.1 | 4.89 | 115.88 |
| Ubiquitin-like modr-activ enzyme 1 | IPI:IPI00123313.1 | 13.99 | 25 | 1058 | 117.7 | 5.66 | 128.44 |
| UDP-gluc:glycoprot glucosyltrans 1 | IPI:IPI00762897.2 | 6.51 | 12 | 1551 | 176.3 | 5.64 | 83.77 |
| Vincµlin | IPI:IPI00405227.3 | 5.63 | 11 | 1066 | 116.6 | 6.00 | 51.27 |
| Xaa-Pro aminopeptidase 1 | IPI:IPI00875027.1 | 12.04 | 17 | 623 | 69.5 | 5.54 | 80.68 |

Table S3 – Biotin-PIF binds the A9 fraction in mouse embryo extracts

| **Description** | **Accession** | **Coverage** | **# Peptides** | **# AAs** | **MW [kDa]** | **calc. pI** | **Score** |
| --- | --- | --- | --- | --- | --- | --- | --- |
| Putative unchar. prot | IPI:IPI00229224.1 | 10.09 | 5 | 109 | 12.5 | 6.52 | 20.22 |
| Tubulin beta-5 chain | IPI:IPI00117352.1 | 2.70 | 3 | 444 | 49.6 | 4.89 | 12.35 |
| Integrin beta-3 | IPI:IPI00266264.1 | 1.91 | 3 | 787 | 86.6 | 5.27 | 11.00 |
| Liver carboxylesterase N | IPI:IPI00138342.3 | 2.17 | 2 | 554 | 61.1 | 5.21 | 9.04 |
| Polyphosphoinositide phospha | IPI:IPI00128101.1 | 1.32 | 1 | 907 | 103.4 | 6.98 | 8.86 |
| IsoLComplement C3 | IPI:IPI00323624.3 | 0.72 | 2 | 1663 | 186.4 | 6.81 | 7.94 |
| Vinculin | IPI:IPI00405227.3 | 1.41 | 2 | 1066 | 116.6 | 6.00 | 7.14 |
| Eef1a1 | IPI:IPI00831184.1 | 5.91 | 2 | 186 | 20.7 | 8.59 | 7.09 |
| Iso-non-muscle myosin alkali L | IPI:IPI00850361.1 | 4.31 | 1 | 116 | 13.0 | 4.65 | 5.77 |
| Putative uncharac protein | IPI:IPI00229080.7 | 1.52 | 3 | 724 | 83.2 | 5.03 | 3.55 |
| Isoform 2 of Myosin-11 | IPI:IPI00227865.1 | 0.77 | 1 | 1938 | 223.1 | 5.47 | 3.06 |

Table S4 (additional proteins identified from Table 4)

| **MISCELLANEOUS** |  |  | First | Second |
| --- | --- | --- | --- | --- |
| Prothymosin alpha | IPI00224784 | 12 kDa | 5 | 4 |
| Calmodulin | IPI00467841 (+1) | 22 kDa | 2 | 5 |
| **Elongation factor 1-alpha 1** | **IPI00307837** | **50 kDa** | **1** | **3** |
| Iso1Plasminogen activator inhibitor 1 RNA-bind | IPI00471475 (+3) | 45 kDa | 2 | 3 |
| Nuclease-sensitive element-BP 1 | IPI00120886 (+1) | 36 kDa | 4 | 3 |
| **Nucleolin** | **IPI00317794** | **77 kDa** | **2** | **5** |
| Granzyme F | IPI00137496 | 28 kDa | 2 | 3 |
| **Endoplasmin** | **IPI00129526** | **92 kDa** | **5** | **10** |
| Desmin | IPI00130102 | 53 kDa | 1 | 3 |
| Isoform 1 of Filamin-A | IPI00131138 (+2) | 281 kDa | 0 | 3 |
| Proteasome subunit alpha type-7 | IPI00131406 | 28 kDa | 1 | 4 |
| Isoform 1 of Glucosidase 2 subunit beta | IPI00115680 (+1) | 59 kDa | 1 | 3 |
| Alpha-2-macroglobulin-P | IPI00454052 | 164 kDa | 0 | 1 |
| Glyceraldehyde-3-phosphate dehydrogenase | IPI00271869 (+11) | 36 kDa | 0 | 3 |
| ATP synthase subunit beta, mitochondrial | IPI00468481 | 56 kDa | 0 | 3 |
| **Serum albumin** | **IPI00131695** | **69 kDa** | **8** | **14** |
| Hemoglobin subunit beta | IPI00762198 (+1) | 16 kDa | 2 | 4 |
| Hemoglobin subunit alpha | IPI00469114 (+1) | 15 kDa | 1 | 2 |
| 60S ribosomal protein L7a | IPI00330363 (+5) | 30 kDa | 4 | 4 |
| 60S ribosomal protein L6 | IPI00313222 (+1) | 34 kDa | 0 | 3 |
| 40S ribosomal protein S25 | IPI00137735 | 14 kDa | 2 | 3 |
| 40S ribosomal protein S3 | IPI00134599 | 27 kDa | 1 | 4 |
| **40S ribosomal protein S3a** | **IPI00331345 (+2)** | **30 kDa** | **1** | **3** |
| 40S ribosomal protein S10 | IPI00112448 | 19 kDa | 0 | 3 |

**Table S5. PepSite 2 prediction of PIF residues participating in targets binding site**

| **PIF targets, binding score and probability** | | | **PIF_1-10_ residues and target residues positions** | | | | | | | | | |
| --- | --- | --- | --- | --- | --- | --- | --- | --- | --- | --- | --- | --- |
| **PDI** | **SCORE** | **P-Value** | **M** | **V** | **R** | **I** | **K** | **P** | **G** | **S** | **A** | **N** |
| 4GSE | 80.10 | 0.003 | met-1::123 |  | arg-3::15 | ile-4::100 |  | pro-6::143 |  |  | ala-9::1 | asn-10::23 |
| 4GSF | 76.90 | 0.005 | met-1::122 | val-2::230 |  | ile-4::91 | lys-5::116 | pro-6::142 |  | ser-8::171 |  |  |
| 4GS8 | 76.70 | 0.005 | met-1::129 |  |  | ile-4::91 | lys-5::116 | pro-6::142 |  |  | ala-9::2 | asn-10::30 |
| 4DTT | 73.70 | 0.009 |  | val-2::225 | arg-3::16 | ile-4::99 |  | pro-6::145 |  |  | ala-9::5 | asn-10::26 |
| 3QZ2 | 73.20 | 0.010 | met-1::129 |  | arg-3::12 |  | lys-5::111 | pro-6::144 |  | ser-8::171 |  | asn-10::30 |
| 3E4A | 72.70 | 0.010 | met-1::130 |  | arg-3::15 | ile-4::97 | lys-5::111 | pro-6::141 |  |  | ala-9::10 |  |
| 3N57 | 72.50 | 0.011 | met-1::128 |  | arg-3::15 | ile-4::100 | lys-5::112 | pro-6::143 |  |  |  | asn-10::27 |
| 3OFI | 72.20 | 0.011 | met-1::123 | val-2::224 | arg-3::20 |  | lys-5::111 |  |  |  | ala-9::1 | asn-10::27 |
| 3CWW | 72.00 | 0.012 |  | val-2::221 | arg-3::13 | ile-4::92 |  |  |  | ser-8::175 | ala-9::1 | asn-10::26 |
| 4DWK | 71.50 | 0.013 | met-1::129 |  | arg-3::16 | ile-4::91 | lys-5::111 |  |  |  | ala-9::2 | asn-10::27 |
| 4GSC | 71.30 | 0.013 | met-1::126 |  | arg-3::11 |  | lys-5::120 | pro-6::142 | gly-7::77 | ser-8::174 |  |  |
| 4IOF | 70.80 | 0.014 |  |  | arg-3::15 |  | lys-5::111 | pro-6::141 | gly-7::76 | ser-8::178 | ala-9::10 |  |
| 2YUQ | 68.90 | 0.019 | met-1::126 | val-2::221 | arg-3::11 | ile-4::99 |  | pro-6::142 |  | ser-8::174 |  |  |
| 2JG4 | 68.90 | 0.020 | met-1::121 | val-2::223 | arg-3::13 |  | lys-5::117 |  |  |  | ala-9::8 | asn-10::25 |
| 2JG4 | 68.90 | 0.020 | met-1::121 | val-2::223 | arg-3::13 |  | lys-5::117 |  |  |  | ala-9::8 | asn-10::25 |
| 3QGW | 68.60 | 0.020 |  |  | arg-3::16 |  |  | pro-6::142 | gly-7::76 | ser-8::174 | ala-9::1 | asn-10::24 |
| 3N56 | 68.40 | 0.021 | met-1::124 |  | arg-3::17 | ile-4::99 |  |  |  | ser-8::173 | ala-9::5 | asn-10::25 |
| 2YPU | 68.00 | 0.022 | met-1::124 | val-2::229 | arg-3::12 |  | lys-5::112 |  |  | ser-8::175 | ala-9::4 |  |
| 1QRQ | 67.80 | 0.023 |  |  | arg-3::14 | ile-4::98 | lys-5::120 | pro-6::150 |  |  |  |  |
| 3HGZ | 62.60 | 0.054 | met-1::125 | val-2::222 | arg-3::15 | ile-4::93 | lys-5::117 |  |  |  |  | asn-10::30 |
| 3V5J | 59.90 | 0.083 | met-1::124 | val-2::223 |  | ile-4::91 | lys-5::111 | pro-6::142 | gly-7::80 |  |  |  |
| 3MJ2 | 59.80 | 0.084 | met-1::128 | val-2::224 | arg-3::12 | ile-4::91 | lys-5::111 | pro-6::141 |  |  |  |  |
| 2LMJ | 59.50 | 0.089 |  |  | arg-3::19 |  | lys-5::119 | pro-6::145 | gly-7::74 | ser-8::173 | ala-9::6 |  |
| 4B8Z | 58.90 | 0.096 | met-1::125 | val-2::228 | arg-3::14 |  |  |  |  |  | ala-9::9 | asn-10::26 |
| 3V8W | 58.50 | 0.102 |  |  | arg-3::11 | ile-4::95 | lys-5::113 | pro-6::142 |  |  | ala-9::10 | asn-10::22 |
| 3MJ1 | 57.70 | 0.115 | met-1::123 |  | arg-3::16 | ile-4::91 | lys-5::113 | pro-6::141 | gly-7::75 |  |  |  |
| 2G54 | 55.00 | 0.170 |  |  | arg-3::18 | ile-4::95 | lys-5::114 | pro-6::141 |  |  | ala-9::10 |  |
| 4HQU | 54.80 | 0.174 | met-1::128 |  | arg-3::18 |  |  | pro-6::144 |  | ser-8::180 | ala-9::3 | asn-10::27 |
| 4HQX | 54.70 | 0.176 | met-1::125 | val-2::226 | arg-3::11 | ile-4::95 | lys-5::111 | pro-6::146 |  |  |  |  |
| 2G49 | 54.60 | 0.179 |  | val-2::224 |  | ile-4::92 | lys-5::118 | pro-6::146 |  | ser-8::179 |  |  |
| 3QGY | 53.90 | 0.197 |  |  | arg-3::12 | ile-4::92 | lys-5::112 | pro-6::148 |  | ser-8::176 | ala-9::10 |  |
| 3V8T | 53.40 | 0.200 |  |  | arg-3::18 | ile-4::96 | lys-5::111 | pro-6::145 | gly-7::74 |  |  | asn-10::24 |
| 2E6I | 53.50 | 0.206 |  | val-2::230 | arg-3::15 | ile-4::93 | lys-5::118 | pro-6::141 |  |  |  |  |
| 3V5L | 53.40 | 0.209 | met-1::124 | val-2::221 | arg-3::15 | ile-4::91 |  | pro-6::145 |  |  |  |  |
| 1PDG | 52.60 | 0.230 | met-1::121 |  | arg-3::15 | ile-4::91 | lys-5::114 | pro-6::147 |  |  |  |  |
| 1PDG | 52.60 | 0.230 | met-1::121 |  | arg-3::15 | ile-4::91 | lys-5::114 | pro-6::147 |  |  |  |  |
| 4B8W | 51.40 | 0.241 | met-1::122 | val-2::225 | arg-3::19 |  |  |  |  |  | ala-9::1 | asn-10::22 |
| 2WBY | 51.90 | 0.250 | met-1::123 |  |  |  | lys-5::112 | pro-6::141 | gly-7::72 |  |  |  |
| 4KIO | 51.80 | 0.250 | met-1::121 | val-2::222 | arg-3::16 | ile-4::95 |  | pro-6::143 |  |  |  |  |
| 2WBY | 51.90 | 0.250 | met-1::123 |  |  |  | lys-5::112 | pro-6::141 | gly-7::72 |  |  |  |
| 3MJG | 50.90 | 0.251 | met-1::122 |  | arg-3::15 | ile-4::94 | lys-5::112 | pro-6::142 |  |  |  |  |
| 2G48 | 50.60 | 0.260 |  | val-2::230 |  |  |  |  | gly-7::77 | ser-8::180 | ala-9::3 | asn-10::22 |
| 1SNX | 46.90 | 0.357 |  |  |  | ile-4::97 | lys-5::115 | pro-6::149 | gly-7::72 | ser-8::171 |  |  |
| 2G47 | 45.10 | 0.412 | met-1::123 |  |  |  |  | pro-6::141 |  | ser-8::172 | ala-9::2 |  |
| 2WK3 | 45.00 | 0.415 |  |  |  | ile-4::91 |  | pro-6::146 | gly-7::75 |  | ala-9::4 |  |
| 3MIY | 44.70 | 0.423 | met-1::121 | val-2::228 | arg-3::15 | ile-4::91 | lys-5::111 |  |  |  |  |  |
| 4HCV | 44.60 | 0.429 |  |  | arg-3::12 | ile-4::95 | lys-5::112 | pro-6::146 |  |  | ala-9::5 |  |
| 2WC0 | 44.10 | 0.445 |  |  |  |  | lys-5::120 |  |  | ser-8::171 | ala-9::3 | asn-10::23 |
| 3H44 | 43.60 | 0.460 | met-1::126 |  |  | ile-4::99 | lys-5::111 | pro-6::150 |  |  |  |  |
| 3E4Z | 40.90 | 0.560 | met-1::121 | val-2::221 |  |  |  |  |  |  | ala-9::1 | asn-10::26 |
| 2G56 | 40.80 | 0.562 | met-1::122 |  |  |  |  | pro-6::142 |  | ser-8::180 | ala-9::5 |  |
| 2JBU | 40.10 | 0.588 | met-1::128 |  |  | ile-4::99 | lys-5::112 | pro-6::148 |  |  |  |  |
| 4HCU | 39.40 | 0.615 | met-1::121 |  | arg-3::20 | ile-4::100 | lys-5::116 |  | gly-7::79 |  |  |  |
| 1SNU | 39.00 | 0.632 | met-1::124 |  |  | ile-4::97 |  | pro-6::141 | gly-7::71 |  |  |  |
| 4E5Y | 38.50 | 0.650 |  |  | arg-3::11 | ile-4::95 |  |  | gly-7::75 | ser-8::172 |  |  |
| 4HCT | 37.20 | 0.699 | met-1::122 |  |  | ile-4::96 | lys-5::112 | pro-6::143 |  |  |  |  |
| 4BKP | 35.20 | 0.772 |  |  |  | ile-4::94 |  | pro-6::141 | gly-7::79 |  |  |  |
| 1SM2 | 35.10 | 0.775 |  |  |  | ile-4::95 |  | pro-6::149 | gly-7::73 | ser-8::175 |  |  |
| 3T9T | 34.30 | 0.803 | met-1::123 |  | arg-3::12 |  |  |  |  |  | ala-9::1 | asn-10::25 |
| 2CRE | 28.10 | 0.954 | met-1::127 |  | arg-3::11 | ile-4::91 | lys-5::116 |  |  |  |  |  |

**Table S6. BeATMuSiC server predicted *in silico* mutagens disrupting the interface of the PIF docking models with several targets**

| **PIF target** | **PDI** | **Highest ∆E [kcal/mol]** | **PIF_1-15_ residues and target residues positions** | | | | | | | | | | | | | | |
| --- | --- | --- | --- | --- | --- | --- | --- | --- | --- | --- | --- | --- | --- | --- | --- | --- | --- |
|  |  |  | **M** | **V** | **R** | **I** | **K** | **P** | **G** | **S** | **A** | **N** | **K** | **P** | **S** | **D** | **D** |
| oxidized PDI, chain A | 4EL1:A | **2.57** |  | **E** |  |  |  | **E** |  |  |  |  |  | **G** |  |  |  |
| oxidized PDI, chain B | 4EL1:B | **3.65** |  |  |  | **G** |  | **G** |  |  |  |  |  |  |  |  |  |
|  |  |  |  |  |  | **D** |  |  |  |  |  |  |  |  |  |  |  |
|  |  |  |  |  |  | **E** |  |  |  |  |  |  |  |  |  |  |  |
|  |  |  |  |  |  | **S** |  |  |  |  |  |  |  |  |  |  |  |
| reduced PDI | 4EKZ | **3.00** |  | **E** |  | **P** |  | **E** |  |  |  |  |  |  |  |  |  |
|  |  |  |  |  |  |  |  | **D** |  |  |  |  |  |  |  |  |  |
|  |  |  |  |  |  |  |  | **S** |  |  |  |  |  |  |  |  |  |
| Potassium Channel Kv Beta-subunit (KCNAB2) | 1ZSX | **3.21** | **A** |  |  |  |  |  |  |  |  |  |  |  |  |  |  |
|  |  |  | **G** |  |  |  |  |  |  |  |  |  |  |  |  |  |  |
| Voltage-dependent K+ channel β-subunit | 1QRQ:A | **4.30** |  |  |  | **G** |  |  |  |  |  |  |  |  |  |  |  |
|  |  |  |  |  |  | **E** |  |  |  |  |  |  |  |  |  |  |  |
|  |  |  |  |  |  | **S** |  |  |  |  |  |  |  |  |  |  |  |
| Voltage-dependent K+ channel β-subunit | 1QRQ:B | **2.99** | **A** |  |  |  |  |  |  |  |  |  |  |  |  |  |  |
|  |  |  | **S** |  |  |  |  |  |  |  |  |  |  |  |  |  |  |
|  |  |  | **G** |  |  |  |  |  |  |  |  |  |  |  |  |  |  |
| Voltage-dependent K+ channel β-subunit | 1QRQ:C | **3.28** |  | **E** |  |  |  |  |  |  |  |  |  |  |  |  |  |
| Voltage-dependent K+ channel β-subunit | 1QRQ:D | **2.36** |  |  |  |  |  | **A** |  |  |  | **P** |  |  |  |  |  |
|  |  |  |  |  |  |  |  | **E** |  |  |  | **G** |  |  |  |  |  |
|  |  |  |  |  |  |  |  |  |  |  |  | **E** |  |  |  |  |  |
| Substrate free IDE – closed conformation | 2JG4:A | **3.03** |  |  |  | **G** |  |  |  |  |  |  |  |  |  |  |  |
|  |  |  |  |  |  | **D** |  |  |  |  |  |  |  |  |  |  |  |
|  |  |  |  |  |  | **E** |  |  |  |  |  |  |  |  |  |  |  |
|  |  |  |  |  |  | **A** |  |  |  |  |  |  |  |  |  |  |  |
|  |  |  |  |  |  | **S** |  |  |  |  |  |  |  |  |  |  |  |
|  |  |  |  |  |  | **K** |  |  |  |  |  |  |  |  |  |  |  |
| Substrate free IDE – closed conformation | 2JG4:B | **2.04** |  |  |  |  | **G** |  |  |  |  |  |  |  |  |  |  |
|  |  |  |  |  |  |  | **D** |  |  |  |  |  |  |  |  |  |  |
| IDE bound to Insulin | 2WBY:A | **3.78** |  |  |  | **G** | **A** |  |  |  |  |  |  |  |  |  |  |
|  |  |  |  |  |  | **G** |  |  |  |  |  |  |  |  |  |  |  |
|  |  |  |  |  |  | **E** |  |  |  |  |  |  |  |  |  |  |  |
|  |  |  |  |  |  | **D** |  |  |  |  |  |  |  |  |  |  |  |
|  |  |  |  |  |  | **S** |  |  |  |  |  |  |  |  |  |  |  |

**Table S7. PIF mutant models**

| **PIF target** | **PDB** | **PIF mutated** | **Mutated Full Sequence** | **Mutation ID** |
| --- | --- | --- | --- | --- |
| red PDI | 4EKZ | P_6_ ->E_6_ | MVRIKEGSANKPSDD | mut1 |
| red PDI | 4EKZ | I_4_ ->P_4_ | MVRPKPGSANKPSDD | mut2 |
| oxid PID, chain A | 4EL1:A | I_4_ -> G_4_ | MVRGKPGSANKPSDD | mut3 |
| oxid PID, chain B | 4EL1:B | V_2_ -> E_2_ | MERIKPGSANKPSDD | mut4 |
